# A “ligand-targeting” peptide-drug conjugate: Targeted intracellular drug delivery by VEGF-binding helix-loop-helix peptides via receptor-mediated endocytosis

**DOI:** 10.1371/journal.pone.0247045

**Published:** 2021-02-25

**Authors:** Masataka Michigami, Kentaro Takahashi, Haruna Yamashita, Zhengmao Ye, Ikuhiko Nakase, Ikuo Fujii

**Affiliations:** 1 Department of Biological Science, Osaka Prefecture University, Sakai, Osaka, Japan; 2 Interprotein Corporation, Osaka, Japan; Synthorx, UNITED STATES

## Abstract

As a new alternative to antibody-drug conjugates, we generated “ligand-targeting” peptide-drug conjugates (PDCs), which utilize receptor-mediated endocytosis for targeted intracellular drug delivery. The PDC makes a complex with an extracellular ligand and then binds to the receptor on the cell surface to stimulate intracellular uptake via the endocytic pathway. A helix-loop-helix (HLH) peptide was designed as the drug carrier and randomized to give a conformationally constrained peptide library. The phage-displayed library was screened against vascular endothelial growth factor (VEGF) to yield the binding peptide M49, which exhibited strong binding affinity (*K*_D_ = 0.87 nM). The confocal fluorescence microscopy revealed that peptide M49 formed a ternary complex with VEGF and its receptor, which was then internalized into human umbilical vein endothelial cells (HUVECs) via VEGF receptor-mediated endocytosis. The backbone-cyclized peptide M49K was conjugated with a drug, monomethyl auristatin E, to afford a PDC, which inhibited VEGF-induced HUVEC proliferation. HLH peptides and their PDCs have great potential as a new modality for targeted molecular therapy.

## Introduction

Antibodies are indisputably the most successful agents used for molecular targeted therapy [1, 2]. However, their use has been limited owing to their bio-physical properties and the cost of manufacture. To enable new applications that overcome some of the limitations of antibody therapeutics, downsized alternative binding molecules have been engineered using natural protein scaffolds and antibody fragments [3, 4]. As an alternative binding molecule with a non-immunoglobulin domain, we have developed a conformationally constrained peptide with a helix-loop-helix (HLH) structure, termed a “molecular targeting HLH peptide” [5–7]. We constructed a phage-displayed peptide library, which was screened against a variety of disease-related proteins to obtain the molecular targeting HLH peptides with antibody-like characteristics in terms of binding affinity and specificity.

The antibody-drug conjugate (ADC) technology is rapidly expanding as a cancer therapeutic approach [8, 9]. However, as with general antibody drugs, ADCs show limitations owing to their large molecular size (~150 kDa): antibodies exhibit poor tumor penetration [10], and induce unwanted immunogenicity unless fully humanized. Especially, antibodies have up to 100 lysine residues so that the drug conjugations yield heterogeneous ADC products [11–13]. Otherwise, the use of site-specific conjugation technologies is required to give homogeneous ADCs [14]. In this work, we examined to expand the drug modality of HLH peptides to peptide-drug conjugates (PDCs) for targeted intracellular drug delivery.

**Fig 1:**
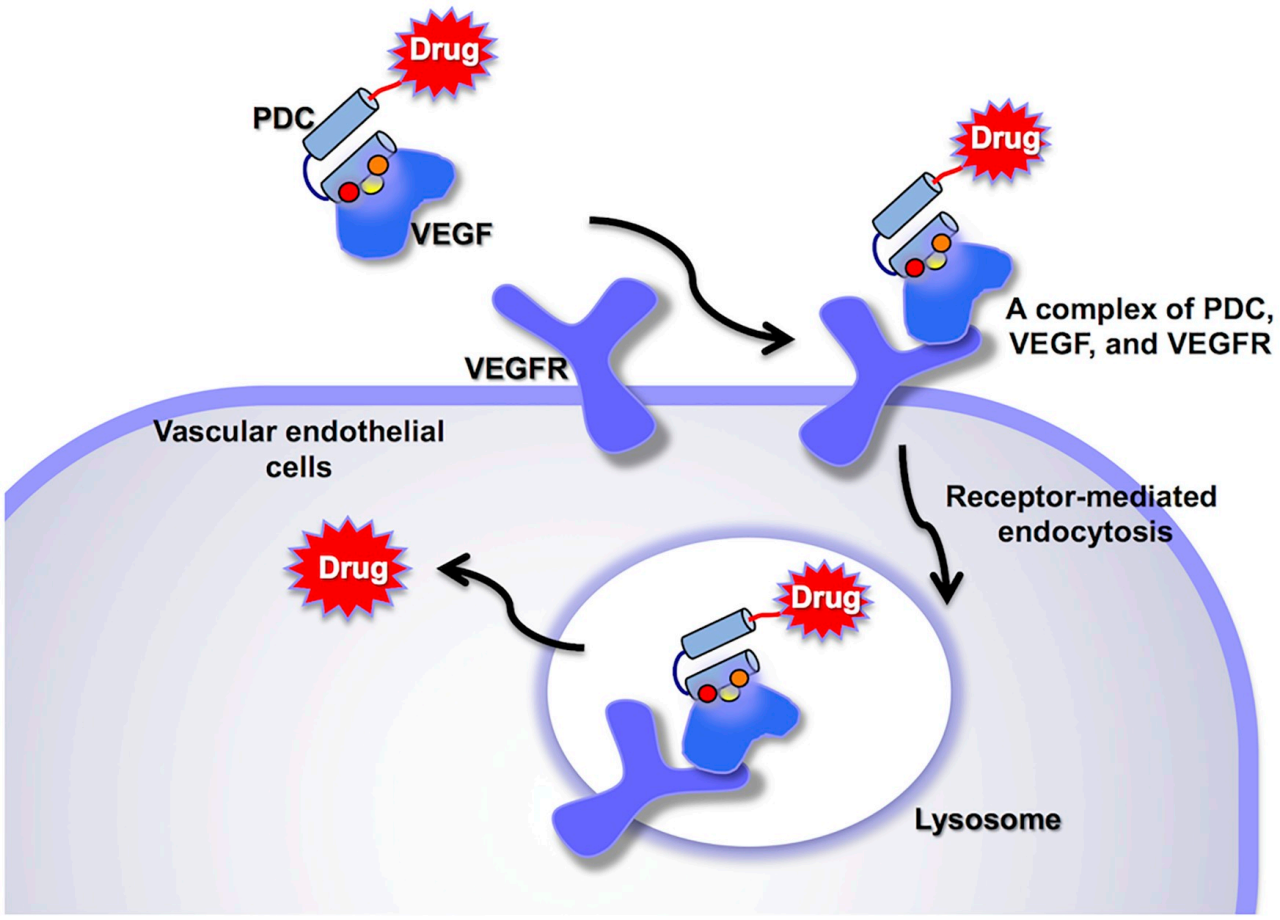
Mode of action of VEGF-targeting Peptide-Drug Conjugates (PDCs). The PDC accumulates around tumor cells, which overexpress VEGF. The PDC-VEGF complex binds to VEGFR on surrounding blood vessels to stimulate receptor-mediated endocytosis, delivering the PDC into the cells. The PDC is broken down in lysosomes to release the drug into the cytosol.

In the ADC mechanism of action, the antibody must bind a cell surface molecule to stimulate the cell internalization by receptor-mediated endocytosis, followed by delivery to lysosomes. However, the requirement for cell internalization makes the generation of ADCs even more complex and difficult [15]. The isolation of antibodies with high cell-internalizing activity requires labor-intensive screening of a massive number of antibodies. Therefore, we designed a "ligand-targeting" peptide-drug conjugate (PDC). As shown in Fig 1, the peptide-ligand complex binds to the receptor to stimulate intracellular uptake via the endocytotic pathway. The small-sized peptides, which bind to the ligand surface adjacent to the receptor-binding site, would not sterically hinder ligand-receptor interactions. Herein, we succeeded in isolating a binding peptide for human vascular endothelial growth factor-A (VEGF-A) from a phage-displayed library of HLH peptides and generating a VEGF-targeting PDC, which was internalized into cells by VEGF receptor (VEGFR)-mediated endocytosis. VEGF-A is a key mediator of tumor angiogenesis. Since VEGF is overexpressed in solid tumors different from VEGFR that is expressed in normal cells [16–19], the PDC would specifically accumulate around tumor cells.

## Results and discussion

### Screening of a phage-displayed HLH peptide library

**Fig 2:**
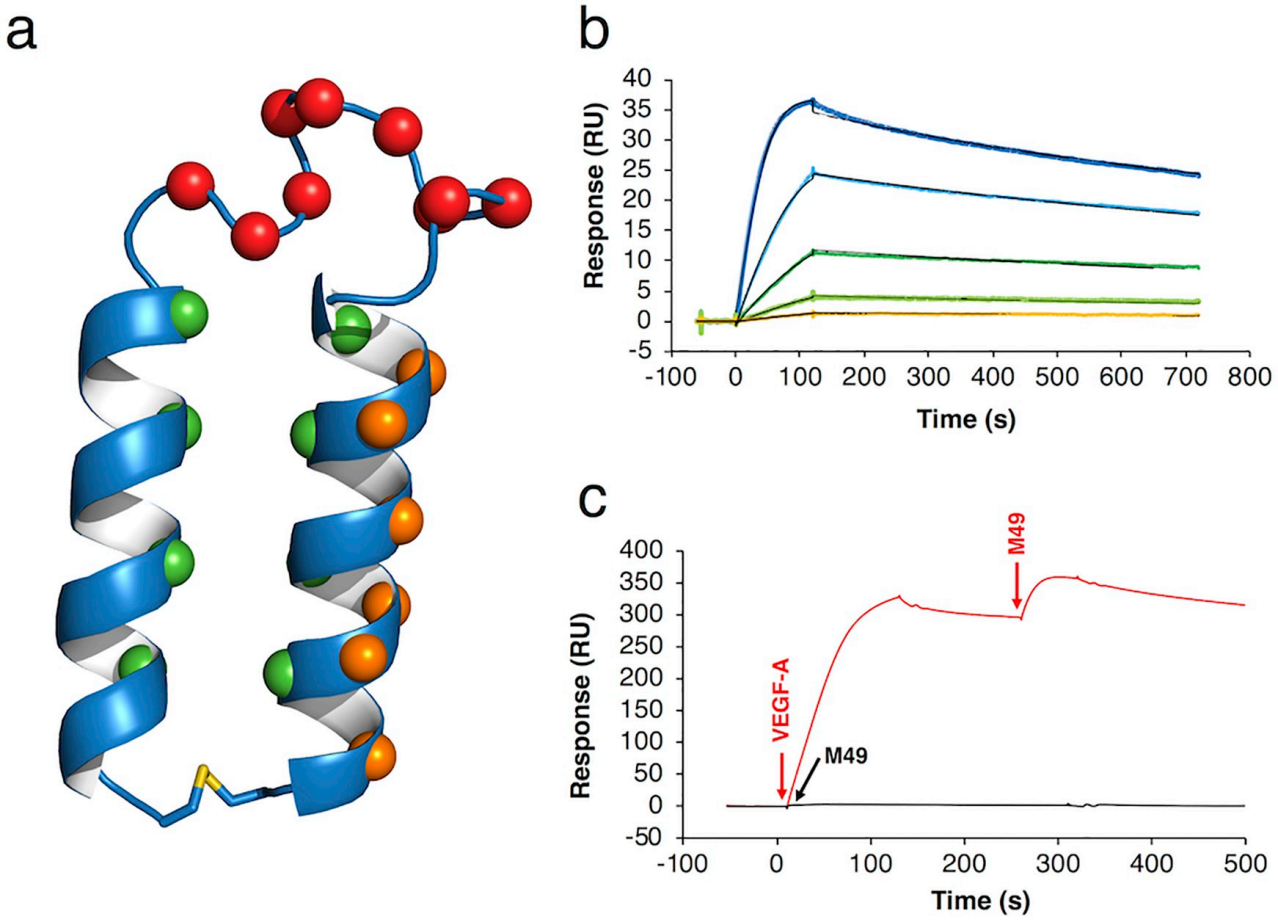
The binding characteristics of M49. (a) Schematic of the HLH peptide library. Spheres colored in green, red, and orange represent the positions of leucines inside of the helices, randomized residues in the loop region, and randomized residues on the C-terminal helix, respectively. (b) The binding affinity of M49 was determined via SPR. VEGF was immobilized by the amine coupling, and the peptides were injected as an analyte at different concentrations (50–3 nM). The data were fitted with the 1:1 Langmuir model. Black lines indicate the fitting curves. (c) Ternary complex formation consisting of M49, VEGF, and VEGFR-2 in the SPR assay. VEGF was injected at a concentration of 25 nM over immobilized VEGFR-2/Fc fusion protein followed by injection of M49 at a concentration of 250 nM (red line). A sensorgram of M49 (250 nM) for VEGFR-2 is shown as a black line. The injection points were represented as arrows.

We constructed a phage-displayed library of molecular targeting HLH peptides. The library scaffold, YT1-S, was designed based on the structural properties of intramolecular antiparallel coiled coils and is composed of three structural regions: the N-terminal α-helix, the C-terminal α-helix, and the connecting loop [3, 20]. In both helical regions, uncharged leucines were incorporated into interhelical positions to dimerize α-helices via hydrophobic interactions. Since the peptide folds by virtue of the hydrophobic core inside of the HLH, solvent-exposed residues could be randomized to yield an HLH peptide library. A library of HLH peptides (CAAELAALEAELAALEGX_1_X_1_X_1_X_1_X_1_X_1_X_1_X_1_X_1_GKLX_2_X_2_LKX_2_KLX_2_X_2_LKX_2_AC) has been displayed on the minor coat protein (pIII) of the M13 filamentous phage by modification of the pComb3 system [21–23]. Oligonucleotides were prepared to randomize 9 positions on the loop region (X_1_, NNK codon, N = A/T/G/C and K = G/C) and the 6 solvent-exposed residues on the C-terminal α-helix (X_2_, NDK codon, D = A/G/T) and were inserted into the M13 phagemid vector. Since Pro breaks the α-helical structure, the NDK codon was used to randomize the C-terminal α-helix to avoid the generation of Pro, Thr, and Ala. Finally, we obtained a phage-displayed HLH peptide library with 1.2 × 10^9^ transformants (Fig 2a).

The phage-displayed peptide library was screened against VEGF-A. Biotinylated VEGF-A was captured using streptavidin-coupled magnetic beads, and VEGF-A-bound phages were eluted with Gly-HCl (pH 2.0). After 4 rounds of biopanning, we observed a strong enrichment of approximately 1,000-fold relative to the background. The isolated clones exhibited specific binding to VEGF-A in phage ELISA, and a majority contained consensus sequences (PWXGYP, PWXG, and DLXXVW, where X is any amino acid) in the loop region, suggesting that the loop region played a role in the binding to VEGF (S1 Table and S1 Fig).

### Binding mode of HLH peptides

Four peptides (M36, M41, M42, and M49) of the identified binding clones were synthesized by standard Fmoc solid-phase peptide synthesis (SPPS). After cleavage from the resin, peptides were cyclized with disulfide bond formation between N- and C-terminal cysteine residues. Interestingly in the cyclization reaction, linear peptides were predominantly converted to cyclic monomers without any detectable oligomers. This result indicates that the linear peptide folds into the HLH structure at neutral pH, and the distance between peptide C- and N-terminals is close enough to promote the intramolecular disulfide bond formation (S2 Fig). All cyclic peptides were evaluated for binding affinity using surface plasmon resonance (SPR), and secondary structures were evaluated using circular dichroism (CD) spectra (S3 and S4 Figs). Peptide M49 was found to have the highest affinity for VEGF-A with a *K*_D_ of 0.87 nM and was subsequently characterized in detail.

As shown in Fig 1, it is essential for the PDC to have binding activity to the complex between VEGF and VEGFR. We therefore examined the binding mode of the HLH peptide by SPR assay (Fig 2c). Recombinant human VEGFR-2 was immobilized on a sensor chip and VEGF-A was captured by VEGFR-2 with a response of 300 RU. Without regeneration of the sensor chip, peptide M49 was injected. As a result, peptide M49 showed a binding response against the VEGF/VEGFR-2 complex, but no response against VEGFR-2 alone. The SPR study revealed that peptide M49, VEGF-A, and VEGFR-2 formed a ternary complex.

### Binding specificity of the HLH peptide M49

**Fig 3:**
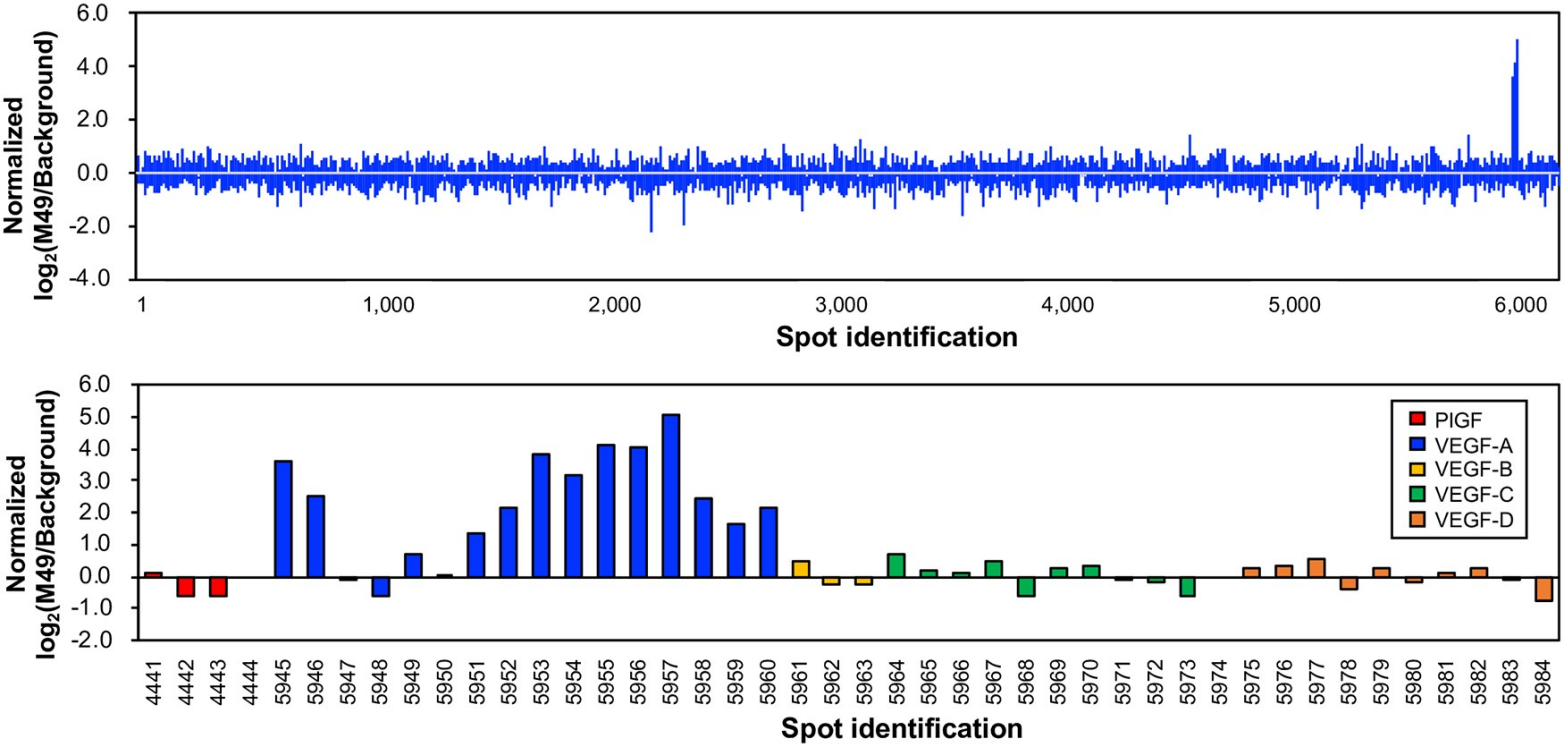
The binding specificity of M49 as revealed by a human proteome microarray. The normalized log_2_(M49/Background) were presented for all 6,144 spots (upper panel) and focusing on VEGF family proteins (lower panel).

Since the molecular size of HLH peptides is extremely smaller than that of antibodies, a point of concern was that the contact area with target proteins would be too small for high-specificity binding. In addition, molecular targeting HLH peptides contain a number of leucine residues that are likely to induce nonspecific hydrophobic interactions. Therefore, we examined the binding specificity of peptide M49 using a human proteome microarray [24, 25]. The microarray contained 6,144 spots of human full-length proteins derived from 2,934 genes. In the assay, M13 phages displaying peptide M49 were incubated with the microarray. After washing, bound phages were detected using an Alexa647-conjugated anti-M13 antibody. Naïve M13 phages with no HLH peptides were assessed in the same manner and treated as background. Consequently, it was found that despite its small molecular size, M49 exhibited extremely high binding specificity for VEGF-A. It is noteworthy that only 10 of 6,144 spots yielded a fluorescence intensity ratio with the normalized log_2_(M49/background) of over 2.0, and all 10 spots were assigned to VEGF-A-immobilized spots (Fig 3 and S1 File). The M49-displaying phages exhibited binding affinity for two VEGF-A isoforms, VEGF-A_121_ and VEGF-A_165_ (S2 Table), but no detectable cross-reactivity was observed for the other VEGF family proteins such as VEGF-B, -C, -D, and placental growth factor (PGF). Some of the VEGF-A-immobilized spots exhibited a low fluorescence intensity ratio, suggesting that the spotted proteins might be denatured. Binding specificity was also confirmed by SPR, showing a binding response to VEGF-A, but not to VEGF-C and -D (S5 Fig).

### Structure-binding relationship and proteolytic resistance of HLH peptide M49

Peptide M49 was designed based on a conformationally constrained HLH scaffold. Therefore, to reveal the importance of the HLH-constrained conformation for binding to VEGF-A, we synthesized M49 derivatives to examine α-helical stability and binding activity (Table 1). A half peptide (loop and C-terminal α-helix) of M49, M49dN, exhibited no α-helical structure and no binding activity. A non-cyclized derivative, M49dS, folded into the helical structure at 20°C and was mostly denatured at 80°C, exhibiting binding activity (*K*_D_ = 27.1 nM). The original cyclic peptide, M49, showed significant structural stability, yielding a typical CD spectrum for the α-helix, even at 80°C (S6A Fig). In addition, we performed a peptide digestion assay of M49 with trypsin because enzymatic degradation is one of the primary pathways for peptide inactivation *in vivo*. The half-lives of M49, M49dS, and M49dN were 1190 ± 60 min, 39 ± 2 min, and 5.0 ± 0.3 min, respectively (S6B Fig). As expected, well-folded peptides exhibited trypsin resistance compared with the unstructured peptide. These peptides have the same number of basic amino acid residues that are cleavage sites for trypsin. It is likely that the difference in tryptic stability is not due to substrate specificity for trypsin, but due to the stability of the peptide conformation. Further, we observed a parallel correlation among peptide structural stability, binding affinity, and proteolytic resistance. The formation of the hydrophobic core inside the two-helix bundle was considered a driving force for folding into the HLH motif. The intramolecular disulfide bond plays an accessory role in structure stabilization.

**Table 1:** Amino acid sequence and dissociation constants of M49 and its variants.

Peptide	Sequence	*K*_D_ (nM)
Library	CAAELAALEAELAALEGX_1_X_1_X_1_X_1_X_1_X_1_X_1_X_1_X_1_KLX_2_X_2_LKX_2_KLX_2_X_2_LKX_2_AC	-
M49	CAAELAALEAELAALEGPWKGYPIPYGKLQFLIKKLKQLKVAC	0.87 ± 0.15
M49dS	AELAALEAELAALEGPWKGYPIPYGKLQFLIKKLKQLKV	27.1 ± 0.4
M49dN	GPWKGYPIPYGKLQFLIKKLKQLKV	non-binding
M49Kmut	*cyclo*(CAELAALEAELAALEGP^18^AKG^21^APIPYGKLQFLIKKLKQLKVAGGGG)	non-binding
Desulfurized M49K	*cyclo*(AAELAALEAELAALEGP^18^WKG^21^YPIPYGKLQFLIKKLKQLKVAGGGG)	4.45 ± 1.30

### Synthesis of HLH peptide M49K as a drug carrier

The molecular targeting HLH peptide M49 has a disulfide bond that is easily reduced *in vivo*. To develop stable PDCs, we improved peptide M49 to enhance its stability against reductants such as glutathione and to introduce accessibility for site-specific drug conjugation. Therefore, the peptide was cyclized by native chemical ligation (NCL), and a free thiol group was introduced into the N-terminus of the peptide for site-specific drug conjugations. We synthesized a backbone-cyclized HLH peptide M49K using Fmoc SPPS (S7 Fig). Fmoc-N-methyl-Cys(Trt)-OH was loaded onto the amide resin for post-SPPS thioesterification [26]. Then, Fmoc-amino acids (5 equiv.) were coupled using DIC/Oxyma (5 equiv.) at room temperature. After cleavage of the peptide from resin, the purified linear peptides were cyclized under NCL conditions (200 mM Na_2_HPO_4_ containing TCEP at pH 7) with 4-mercaptophenylacetic acid (MPAA) as a catalyst [27] to yield the cyclized peptide M49K. To determine its physicochemical properties, M49K was subjected to desulfurization [28] to prevent the formation of the disulfide dimer during measurements. The desulfurized peptide M49K displayed an α-helical structure in 20 mM phosphate buffer (pH 7.0) at 20°C and a strong binding response with a *K*_D_ of 4.45 ± 1.30 nM (S8 Fig).

### Cellular uptake

**Fig 4:**
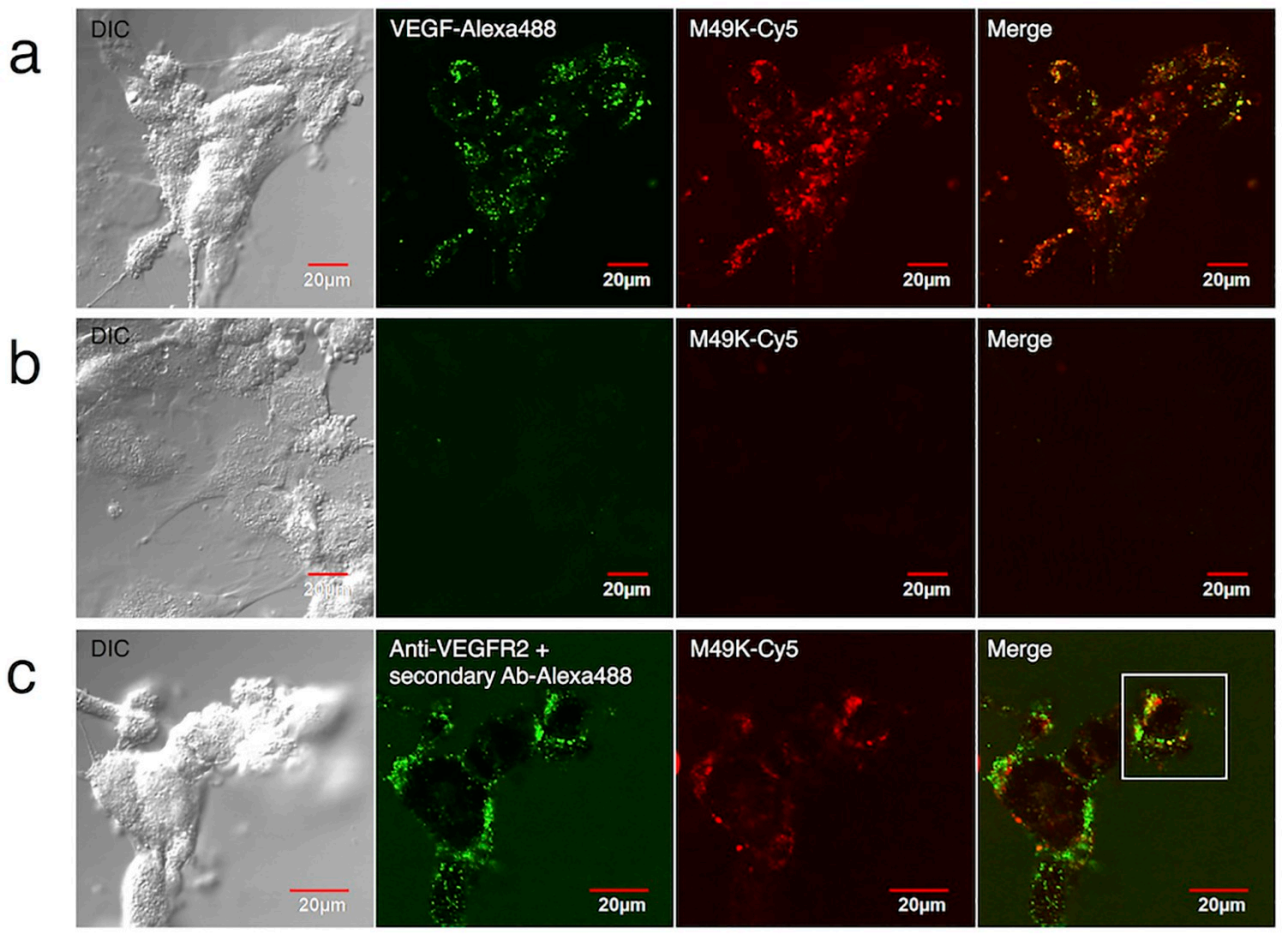
Confocal microscopy of HUVECs treated with Cy5-labeled M49K. HUVECs were incubated with different solutions for 6h at 37°C before imaging: (a) M49K-Cy5 and VEGF-Alexa488 conjugate and (b) M49K-Cy5. (c) At 4°C, HUVECs were treated with M49K-Cy5 and VEGF, and VEGFR2 was detected using a mouse anti-VEGFR2 antibody and an Alexa488-anti-mouse IgG antibody. The white square has been enlarged in S10B Fig.

Next, we examined the cellular uptake ability of M49K via VEGFR-mediated endocytosis by confocal microscopy. Cy5-maleimide was selectively linked to a thiol group on M49K (S9 Fig). An Alexa Fluor 488 succinimidyl ester was coupled to recombinant human VEGF-A_165_, followed by purification using a desalting spin column. HUVECs were treated with 100 nM Cy5-labeled M49K for 6 h in the presence of 50 nM of the VEGF-Alexa488 conjugate. The fluorescence signal of M49K-Cy5 was observed in endosomal vesicles and overlapped with that of VEGF-Alexa488 (Fig 4a and S10A Fig). No Cy5 signal was detected in the absence of VEGF-Alexa488 (Fig 4b). Furthermore, we examined the formation of the ternary complex of M49, VEGF-A, and VEGFR on the cell surface of HUVECs under the condition that no endocytosis would occur (Fig 4c). After HUVECs were incubated with M49K-Cy5 and VEGF-A for 1 h at 4°C, VEGFR was detected through a combination of anti-VEGFR-2 antibody and Alexa488-anti-mouse IgG antibody. Confocal microscopy revealed M49K and VEGFR-2 co-localization on cell surfaces and no signals of internalized M49K-Cy5 inside HUVECs. These results suggested a cellular uptake mechanism in which M49K, VEGF-A, and VEGFR form a ternary complex on the cell surface to initiate receptor-mediated endocytosis, which was also supported by flow cytometric analysis (S11 Fig).

### Cell growth inhibition

**Fig 5:**
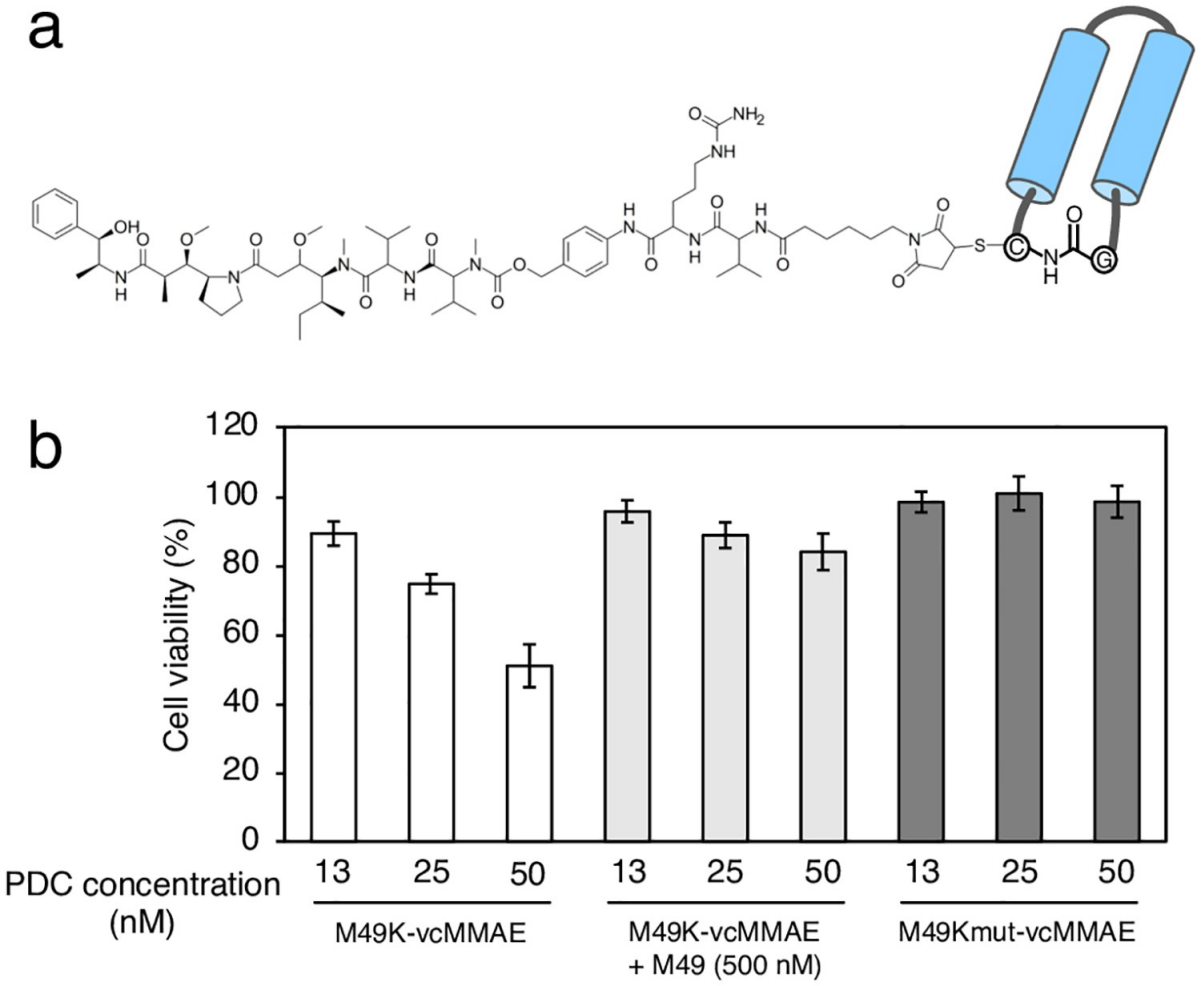
Peptide-drug conjugate structure and cell viability. (a) Structure of peptide-drug conjugate M49K-vcMMAE. (b) Cell viability assay. HUVECs were treated with VEGF (25 ng/mL) and peptide-drug conjugates. After 24 h, cell viability was measured using WST-1 reagent. The data represent the mean ± standard deviation (n = 3).

Finally, we synthesized a PDC consisting of M49K and monomethyl auristatin E (MMAE) to evaluate its inhibitory activity on HUVEC proliferation (Fig 5a). MMAE, a potent microtubule inhibitor, was linked to a thiol group of M49K using a maleimidocaproyl-valine-citrulline-*p*-aminobenzoyloxy-carbonyl linker to obtain the PDC, M49K-vcMMAE (41% yield), which exhibited a binding affinity with a *K*_D_ of 30 nM (S12 Fig). The valine-citrulline dipeptide in this linker is cleavable by cathepsin B to facilitate drug release inside cells [29]. In the cell-based assay, the viability of HUVECs in response to VEGF-A was set to 100%. The addition of M49 had no effect on VEGF-induced HUVEC proliferation, suggesting that M49 had no effect on VEGF/VEGF interaction (S13 Fig). The PDC, M49K-vcMMAE, decreased HUVEC viability with an IC_50_ of 50 nM. As shown in Fig 5b, the decrease in cell viability causing by M49K-vcMMAE was reversed by the addition of excess M49, which competed with the PDC for VEGF-A binding. In contrast, M49Kmut-vcMMAE, an M49K mutant (Trp18Ala and Tyr21Ala) conjugated to MMAE with no affinity for VEGF-A, did not inhibit HUVEC proliferation. From these findings, we concluded that M49K-vcMMAE was internalized into HUVECs via VEGF-A/VEGFR-mediated endocytosis and successfully delivered the cytotoxic drug into cells, thus inhibiting cell growth.

## Conclusions

In this work, a phage-displayed library of *de novo* designed HLH peptides was constructed and screened against VEGF-A. We successfully demonstrated that the most potent HLH peptide, M49, formed a ternary complex with VEGF-A and VEGFR, and was then internalized into HUVECs via receptor-mediated endocytosis. Finally, peptide M49 was conjugated to MMAE to yield a VEGF-targeting PDC, which was also internalized into HUVECs to inhibit the cell growth. These results suggest that VEGF-targeting PDCs have the potential to deliver cytotoxic drugs into the tumor vasculature. VEGF is a well-established therapeutic target for cancer disease. VEGF-targeted therapeutics containing neutralizing antibodies (bevacizumab, ramucirumab) [30, 31], soluble VEGFR (aflibercept) [32], and small-molecule tyrosine kinase inhibitors (sunitinib, pazopanib) [33, 34] have been designed to block the VEGF signaling to give anti-angiogenic activity. Differing from the mode of action of conventional VEGF inhibitors, PDCs deliver cytotoxic drugs inside cells to cause the arrest of cell division. Moreover, PDCs would target cancer cells, since autocrine/paracrine VEGF signaling occurs within various cancer cell types [35–38].

Notably, to the best of our knowledge, molecular targeting HLH peptide M49 is the most potent binding peptide against VEGF-A (*K*_D_ = 0.87 nM). Furthermore, M49 showed a high target specificity for VEGF-A, as confirmed by the human proteome microarray, and strong proteolytic resistance. The high potency of M49 can be attributed to its constrained conformation of the α-helical structure. The pre-organized constrained conformation limited unfavorable entropy loss occurring during the interaction of M49 with VEGF-A, thus resulting in the strong binding affinity. The constrained conformation also limited adoption of the binding conformation to other proteins and to the active sites of any proteases.

One of the major difficulties in ADC development is to generate antibodies that efficiently internalize into cells. In many cases, after screening for antibodies that bind to cell surface receptors based on their target binding affinity, subsequent screening will be required to discover the internalizing antibodies among them. Herein, we demonstrated a strategy based on "ligand-targeting" PDC for intracellular delivery. Thus, unlike ADCs, the PDCs require no extensive screening to obtain internalizing peptides, since the PDCs utilize natural ligand-receptor interactions inducing endocytosis for the internalization. The molecular targeting HLH peptides occupy a unique “middle ground” between small-molecule drugs and antibody drugs. These mid-sized peptides are expected to comprise a next-generation therapeutic modality as non-antibody scaffolds, facilitating future research in chemical biology and drug development.

## Methods

### Biopanning

Recombinant human VEGF-A_165_ (R&D systems) was biotinylated by using an EZ-Link Sulfo-NHS-Biotin Kit (ThermoFisher Scientific) by mixing a 1:50 molar ratio of VEGF-A_165_ to Sulfo-NHS-Biotin and incubated for 2 hours on ice. The biotinylated VEGF was purified using a Zeba Spin Desalting Column, 7K MWCO, 0.5 mL (ThermoFisher Scientific). The phage library was incubated with 100 nM of biotinylated VEGF-A_165_ in 3% BSA-containing PBS. VEGF-bound phages were captured with Dynabeads M-280 Streptavidin (Invitrogen). After washing with PBST (PBS with 0.05% Tween20), VEGF-bound phages were eluted with 200 μL of 0.2 M Gly-HCl (pH 2.0) for 10 min and neutralized with 20 μL of 2 M Tris immediately. Recovered phages were amplified with XL1-Blue cells for the subsequent round of screening.

### Phage ELISA

For protein immobilization, 50 μL of VEGF-A solution (10 μg/mL) and BSA solution (10 μg/mL) were dispensed to ELISA plates (POLYSORP, Nunc) in carbonate buffer (pH 8.0). After the incubation at 4°C for 12 h, we performed washing and blocking with SuperBlocking Buffer (Thermo scientific). The number of 2.5 x 10^9^ phages were added to each well, and binding phages were detected using an anti-M13 antibody-HRP conjugate.

### Fmoc solid-phase peptide synthesis

The peptides were synthesized manually by SPPS using the Fmoc strategy on a Fmoc-SAL-PEG-PS resin (substitution: 0.23 mmol/g). Fmoc deprotection was performed 20% PPD/DMF (1×1 min, 1×15 min), followed by coupling of amino acids (5 equiv.) using DIC (5 equiv.) and Oxyma (5 equiv.) in DMF (60 min). To avoid the formation of deletion peptides, acetylation with 10% Ac_2_O/DMF (10 min) after the coupling step was performed. The assembled peptides were deprotected and cleaved from the solid support with cleavage cocktail containing TFA/H_2_O/TIS/EDT (94/2.5/1/2.5) at room temperature for 2 hours. Three rounds of extraction of the peptides were carried out using ice-cold diethyl ether. Crude peptides were analyzed and purified by RP-HPLC system (Hitachi) with a C18 column (250 × 10 mm, 5 μm, YMC) using linear gradients of 30 to 60% B (A: 0.1% TFA in water, B: 0.08% TFA in acetonitrile) over 30 min at a flow rate of 3.0 mL/min. Fractions were analyzed with MALDI-TOF-MS (AoutflexII, Bruker Daltnics). The disulfide bond formation was performed following procedures. The thiol free peptide (5 mg) was dissolved in 50 mL of 20 mM NH_4_HCO_3_ (pH 8.0) and stirred for 12 hours. After the reaction, the solution was lyophilized and purified by RP-HPLC. All peptides were obtained with a purity >95%.

### Surface plasmon resonance

Binding assays were performed with a BiacoreT200 instrument (GE Healthcare) using HBS-EP+ buffer (25 mM HEPES, pH 7.4, 150 mM NaCl, 3 mM EDTA, 0.05% surfactant P20). Recombinant human VEGF-A_165_ was immobilized on a CM5 sensor chip using the standard amine coupling procedure. In the kinetics assay of M49, the immobilization level of VEGF-A_165_ was controlled to reach approximately 100 RU, providing a maximum M49 response of 40 RU. Data were analyzed by Biacore T200 Evaluation Software (GE Healthcare).

### Human proteome microarray

We evaluated the peptide binding specificity by using a human proteome microarray (Fukushima Translational Research Project, Japan). The microarrays were treated with a blocking buffer and incubated with Goat Reference Antibody Mixture I (Fukushima Protein Factory, Inc., Japan) and M49 peptide displaying M13 phages. After the incubation for 17 hours at 37°C, microarrays were double-labeled with Alexa Fluor 647-conjugated anti-M13 major coat protein antibody (RL-ph1, Santa Cruz Biotechnology, Inc., USA) and Cy3-conjugated anti-goat IgG antibody. The stained microarrays were scanned with a GenePix 4000B scanner. We normalized the fluorescence intensity of Alexa Fluor 647 by that of Cy3 to compare the peptide binding signals between each microarray because the fluorescence intensity of Cy3 correlates with the quantity of the spotted protein. Then, we calculated data by subtracting the values of backgrounds to remove the non-specific binding of the secondary antibodies and M13 phages.

### Backbone cyclization of M49 derivatives by NCL

The C-terminal thioesterification and NCL reaction were performed simultaneously. A linear peptide (10 mg) was dissolved in 1 mL of 200 mM Na_2_HPO_4_ containing 20 mM TCEP and 2 mM MPAA at pH 7. The reaction proceeded for 12 hours at room temperature. After the reaction, the cyclized peptide was purified by RP-HPLC and lyophilized (55% yield). Molecular mass was confirmed by MALDI-TOF-MS. Calcd for C_222_H_355_N_54_O_57_S [M+H]^+^: 4724.69 (average isotopes), Found [M+H]^+^ m/z = 4724.18.

### Desulfurization of M49K peptide

Peptide (5.0 mg) was dissolved in 1 mL of 100 mM Tris-HCl (pH 6.5) containing 200 mM VA-044, 250 mM TCEP, and 40 mM glutathione. The reaction was performed for 12 hours at room temperature. The desulfurized peptide was purified by RP-HPLC and lyophilized (3.3 mg, 65% yield). Molecular mass was confirmed by MALDI-TOF-MS. Calcd for C_222_H_355_N_54_O_57_ [M+H]^+^: 4692.52 (average isotopes), Found [M+H]^+^ m/z = 4691.09.

### CD spectroscopy

CD spectra between 260 and 190 nm were collected with a J-820 spectropolarimeter (Jasco, Japan) by using a 0.1 cm path length quartz cell. The peptides were dissolved in 20 mM phosphate buffer (pH 7.0) at 20 μM of concentration. The scan speed, response time, and bandwidth for J-820 were 50 nm/min, 2 s, and 1 nm, respectively.

### Tryptic stability

Each peptide was dissolved in 100 mM Tris-HCl (pH 8.5) at a concentration of 300 μM, and trypsin was also dissolved in the 100 mM Tris-HCl buffer at a concentration of 75 nM. For stability assay, 100 μL of the peptide solution and 100 μL of freshly prepared trypsin solution were mixed and incubated at 37°C. Aliquots of 20 μL were sampled at different time intervals and mixed with 60 μL of 1M HCl. The remaining peptide was analyzed by RP-HPLC.

### Synthesis of M49K-Cy5

M49K was dissolved in water at a concentration of 3 mM, and Cy5-Maleimide was dissolved in DMF at a concentration of 15 mM. M49K (15 μL, 45 nmol, 1 equiv.) and Cy5-Maleimide (30 μL, 450 nmol, 10 equiv.) were mixed and incubated for 12 hours at room temperature in the dark. Cy5-labeled M49K was purified RP-HPLC and lyophilized. Molecular mass was confirmed by MALDI-TOF-MS. Calcd for C_261_H_400_N_58_O_66_S_3_ [M+H]^+^: 5501.51 (average isotopes), Found [M+H]^+^ m/z = 5501.08.

### Confocal microscopic observation

HUVECs were expanded in EGM-2 medium (Lonza) and seeded on 35 mm glass-based dish (IWAKI) and cultured in DMEM supplemented with 10% FCS for 12 hours. After 5 times wash with PBS, each peptide sample and VEGF-Alexa488 were diluted in EBM-2 and added into HUVECs coated glass-based dish for 2 hours in a CO_2_ incubator. For VEGFR co-localization assay, HUVECs were treated with a mixture of M49K-Cy5 (500 nM) and recombinant human VEGF-A_165_ (100 nM) for 1 hour at 4°C. After the wash with cold PBS, VEGFR-2 was stained by the combination of mouse anti-VEGFR2 antibody (ab9530, Abcam) and Alexa Fluor goat anti-mouse IgG (H+L) (Invitrogen) at 4°C. Confocal images were collected using an FV1200 laser scanning microscope (Olympus).

### Flow cytometric analysis

HUVECs were seeded in gelatin-coated 24-well microplate in EBM-2 supplemented with 2% FCS. After overnight incubation, HUVECs were treated with VEGF-Alexa488 (100 nM) and/or M49K-Cy5 (500 nM) for 6 hours in a CO_2_ incubator. After 5 times wash with PBS, HUVECs were trypsinized for 10 min to detach from a culture dish and remove the VEGF that bound to the cell surface. The trypsinized HUVECs were neutralized using DMEM medium and analyzed by flow cytometer (BD Accuri C6).

### Synthesis of M49K-vcMMAE

M49K (3 mg, 0.6 μmol, 1 equiv.) and maleimide-functionalized vcMMAE (0.9 mg, 0.7 μmol, 1.1 equiv.) were dissolved in 1 mL of DMF. The reaction was performed for 12 hours at room temperature. The reaction product, M49K-vcMMAE, was purified by RP-HPLC and lyophilized (white powder, 1.6 mg, 41% yield). Molecular mass was confirmed by MALDI-TOF-MS.

### VEGF-induced HUVEC proliferation assay

HUVECs (5000 cells/100 μL/well) were seeded in gelatin-coated 96-well microplate in EBM-2 supplemented with 2% FCS. After overnight incubation, HUVECs were stimulated by 25 ng/mL of VEGF-A_165_ in the presence of M49Kmut-vcMMAE (50 nM), M49K-vcMMAE (50 nM), and the mixture of M49K-vcMMAE (50 nM) and M49 (500 nM) for 24 hours at 37°C. Then, 10 μL of WST-1 reagent was added into each well. After 1 hour of incubation at 37°C, absorbance was read at 450 nm. The control condition for 100% proliferation was obtained from wells coated with VEGF stimulated HUVECs, and 0% was obtained from wells without cells. Data represent mean ± standard deviation of three independent experiments.

## Supporting information

S1 File Microarray data. The binding signals of M49 against all spots were presented. Normalized log_2_(M49/background) was calculated using the fluorescence intensity of anti-goat IgG-Cy3 conjugate and anti-M13 antibody-Alexa647 conjugate that were indicated as green intensity and red intensity, respectively.
(XLSX)

S1 Fig Phage ELISA analysis of the selected 10 clones from the biopanning. The all clones specifically bound to VEGF-A, and M49 phage clone presented the strongest binding signal to VEGF-A. The data represent the mean ± standard deviation (n = 3).
(TIF)

S2 Fig The cyclization of HLH peptide M49 by a disulfide bond. The reaction was monitored by RP-HPLC. The UV profile at 280 nm is shown. The separations were performed using a linear gradient (10–90%) of eluent B in eluent A over 30 min (eluent A = 0.1% TFA in water; eluent B = 0.08% TFA in acetonitrile). Linear M49 was converted to cyclic M49 (MALDI-TOF-MS: observed [M+H]^+^ 4682.1 (m/z), calculated [M+H]^+^ using monoisotopes 4682.6).
(TIF)

S3 Fig CD spectra of VEGF-targeting HLH peptides. CD spectra were collected at 20°C in PBS, and the concentration of peptides was 20 μM. The synthetic peptides showed typical α-helical arrangement (negative maxima at 222 and 208 nm, positive maximum at approximately 190 nm).
(TIF)

S4 Fig The binding affinity of VEGF-targeting HLH peptides. The binding affinity of (A) M36, (B) M41, (C) M42 and (D) M49 were determined with SPR. The sensorgrams and scatchard plots were presented. *K*_D_ values (n = 3) were 96.9 ± 3.0 nM, 1960 ± 20 nM, 3540 ± 80 nM and 0.87 ± 0.15 nM, respectively. Sensorgrams of M49 were fitted with the 1:1 Langmuir model. *k*_a_, (9.2 ± 1.9) × 10^5^ (1/Ms); *k*_d_, (7.6 ± 0.1) × 10^−4^ (1/s).
(TIF)

S5 Fig The binding specificity of M49 for VEGF family proteins. VEGF-A, -C, and -D were immobilized on CM5 sensor chip at 1000 RU by using amine coupling method. (A) The sensorgrams of peptide M49 to immobilized VEGF proteins. Peptide M49 was injected at a concentration of 1 μM, and association time was 60 seconds. (B) The sensorgrams of bevacizumab at a concentration of 100 nM.
(TIF)

S6 Fig Structure-activity relationship of M49. (A) CD spectra of peptide M49 and its variants at 20°C (solid line) and 80°C (dash line). (B) Tryptic stability of M49 peptide and its variants. The peptides were incubated with trypsin, and the remaining peptides were analyzed by RP-HPLC. The data represent the mean ± standard deviation (n = 3). (C) The amino acid sequence of the synthetic peptides. Underlined cysteine residues are involved in disulfide bond formation. These peptides were synthesized using Fmoc-SPPS and molecular mass was confirmed by using MALDI-TOF-MS.
(TIF)

S7 Fig Synthesis of the backbone-cyclized helix-loop-helix peptide. (a) 94:2.5:2.5:1 TFA/EDT/H_2_O/TIS, 3h, 15% yield; (b) 2 mM MPAA, 20 mM TCEP, 200 mM Na_2_HPO_4_ pH7, 12h, RT, 40% yield; (c) 200 mM VA-044, 250 mM TCEP, 40 mM Glutathione, 12h, RT, 65% yield.
(TIF)

S8 Fig Secondary structure and binding activity of desulfurized M49K. (A) CD spectra of M49 and desulfurized M49K at 20°C in 20 mM phosphate buffer (pH 7.0). (B) Sensorgrams of desulfurized M49K binding to recombinant human VEGF-A_165_. M49K (200–6 nM) were injected over the VEGF-A immobilized flow cell as an analyte. Flow rate, temperature, and running buffer were 3.0 μL/min, 25°C, and HBS-EP+, respectively. The binding parameters were calculated by BiacoreT200 evaluation software with the 1:1 Langmuir model. The fitting curves were indicated as black lines. *K*_D_, 4.45 ± 1.30 (nM); *k*_a_, (4.46 ± 0.71) × 10^5^ (1/Ms); *k*_d_, (2.02 ± 0.75) × 10^−3^ (1/s). The data represent the mean ± standard deviation (n = 3).
(TIF)

S9 Fig Purity and binding affinity of M49K-Cy5. (A) Analytical HPLC profile of M49K-Cy5. Analytical HPLC was performed linear gradient (10–90%) of eluent B in eluent A over 30 min. (eluent A = 0.1% TFA in water; eluent B = 0.08% TFA in acetonitrile) on a C-18 column (250 × 4.6 mm, YMC-Pack). (B) MALDI-TOF-MS spectra of M49K-Cy5. Calculated for C_261_H_400_N_58_O_66_S_3_ [M+H]^+^: 5501.51 (average isotopes), Found [M+H]^+^ m/z = 5501.08 (C) Sensorgram of the M49K-Cy5 binding to VEGF-A165. M49K-Cy5 was injected over the VEGF immobilized flow cell as an analyte (400–25 nM) at 25°C. The binding parameters were calculated by BiacoreT200 evaluation software with the 1:1 Langmuir model. The fitting curves were indicated as black lines and *K*_D_ value was 5.5 nM.
(TIF)

S10 Fig Co-localization analysis of peptide M49K in confocal microscopy. (A) HUVECs were treated with M49K-Cy5 (500 nM) and VEGF-Alexa488 (100 nM) for 6 hours at 37°C. Cell nuclei were stained by Hoechst 33342 before imaging. White arrows indicate co-localized vesicles. (B) An enlarged image of Fig 3c in the main text.
(TIF)

S11 Fig Peptide cellular uptake in HUVECs using flow cytometric analysis. HUVECs were incubated for 6 hours with different solutions: VEGF-Alexa488 (100 nM), M49K-Cy5 (500 nM), and mixture of VEGF-Alexa488 (100 nM) and M49K-Cy5 (500 nM). PBS treated cells served as negative control.
(TIF)

S12 Fig The purity and binding activity of M49K-vcMMAE (left panels) and M49Kmut-vcMMAE (right panels). (A) Analytical HPLC profile of purified PDCs. Analytical HPLC was performed linear gradient (10–90%) of eluent B in eluent A over 30 min. (eluent A = 0.1% TFA in water; eluent B = 0.08% TFA in acetonitrile) on a C-18 column (250 × 4.6 mm, YMC-Pack). (B) MALDI-TOF-MS spectra of PDCs. M49K-vcMMAE: Calculated for C_290_H_461_N_65_O_72_S [M+H]^+^: 6042.22 (average isotopes), Found [M+H]^+^ m/z = 6041.81, M49Kmut-vcMMAE: Calculated for C_276_H_452_N_64_O_71_S [M+H]^+^: 5834.99 (average isotopes), Found [M+H]^+^ m/z = 5833.86 (C) Sensorgrams of the PDCs binding to VEGF-A_165_. M49K-vcMMAE was injected over the VEGF immobilized flow cell as an analyte (1000–16 nM). The binding parameters were calculated by BiacoreT200 evaluation software with the 1:1 Langmuir model. The fitting curves were indicated as black lines. *K*_D_ value was 30 nM. M49Kmut-vcMMAE was injected at a concentration of 1000 nM.
(TIF)

S13 Fig Cell viability in HUVECs treated with M49 and M49K-vcMMAE. The IC_50_ value of M49K-vcMMAE is 50 nM. The data represent the mean ± standard deviation (n = 3).
(TIF)

S1 Table Amino acid sequence of the peptides selected from the biopanning. After 4 rounds of the screening, we obtained 10 HLH peptide clones. The consensus sequences were displayed in red, and the numbers of identified clones were showed as frequency.
(TIF)

S2 Table VEGF-A proteins spotted on the protein microarray. (TIFF)

## References

[1] Urquhart L. Top companies and drugs by sales in 2019. Nat Rev Drug Discov. 2020;19(4):228. 10.1038/d41573-020-00047-7 . 32203287

[2] Kaplon H, Muralidharan M, Schneider Z, Reichert JM. Antibodies to watch in 2020. MAbs. 2020;12(1):1703531. 10.1080/19420862.2019.1703531 . 31847708 PMC6973335

[3] Holliger P, Hudson PJ. Engineered antibody fragments and the rise of single domains. Nat Biotechnol. 2005;23(9):1126–1136. 10.1038/nbt1142 . 16151406

[4] Binz HK, Amstutz P, Plückthun A. Engineering novel binding proteins from nonimmunoglobulin domains. Nat Biotechnol. 2005;23(10):1257–1268. 10.1038/nbt1127 . 16211069

[5] Suzuki N, Fujii I. Optimazation of the loop length for folding of a helix-loop-helix peptide. Tetrahedron Lett. 1999;40(13):6013–6017

[6] Fujiwara D, Kitada H, Oguri M, Nishihara T, Michigami M, Shiraishi K, et al. A Cyclized Helix-Loop-Helix Peptide as a Molecular Scaffold for the Design of Inhibitors of Intracellular Protein-Protein Interactions by Epitope and Arginine Grafting. Angew Chem Int Ed Engl. 2016;55(36):10612–10615. 10.1002/anie.201603230 . 27467415

[7] Ramanayake Mudiyanselage TMR, Michigami M, Ye Z, Uyeda A, Inoue N, Sugiura K, et al. An Immune-Stimulatory Helix-Loop-Helix Peptide: Selective Inhibition of CTLA-4-B7 Interaction. ACS Chem Biol. 2020;15(2):360–368. 10.1021/acschembio.9b00743 . 31841301

[8] Beck A, Goetsch L, Dumontet C, Corvaïa N. Strategies and challenges for the next generation of antibody-drug conjugates. Nat Rev Drug Discov. 2017;16(5):315–337. 10.1038/nrd.2016.268 . 28303026

[9] Perez HL, Cardarelli PM, Deshpande S, Gangwar S, Schroeder GM, Vite GD, et al. Antibody-drug conjugates: current status and future directions. Drug Discov Today. 2014;19(7):869–881. 10.1016/j.drudis.2013.11.004 . 24239727

[10] Saga T, Neumann RD, Heya T, Sato J, Kinuya S, Le N, et al. Targeting cancer micrometastases with monoclonal antibodies: a binding-site barrier. Proc Natl Acad Sci U S A. 1995;92(19):8999–9003. 10.1073/pnas.92.19.8999 . 7568060 PMC41095

[11] Jain N, Smith SW, Ghone S, Tomczuk B. Current ADC Linker Chemistry. Pharm Res. 2015;32(11):3526–3540. 10.1007/s11095-015-1657-7 . 25759187 PMC4596905

[12] Kim MT, Chen Y, Marhoul J, Jacobson F. Statistical modeling of the drug load distribution on trastuzumab emtansine (Kadcyla), a lysine-linked antibody drug conjugate. Bioconjug Chem. 2014;25(7):1223–1232. 10.1021/bc5000109 . 24873191

[13] Wang L, Amphlett G, Blättler WA, Lambert JM, Zhang W. Structural characterization of the maytansinoid-monoclonal antibody immunoconjugate, huN901-DM1, by mass spectrometry. Protein Sci. 2005;14(9):2436–2446. 10.1110/ps.051478705 . 16081651 PMC2253466

[14] Zhou Q. Site-specific conjugation for ADC and beyond. Biomedicines 2017;5(4):64–78. 10.3390/biomedicines5040064 . 29120405 PMC5744088

[15] Gerber HP, Kung-Sutherland M, Stone I, Morris-Tilden C, Miyamoto J, McCormick R, et al. Potent antitumor activity of the anti-CD19 auristatin antibody drug conjugate hBU12-vcMMAE against rituximab-sensitive and -resistant lymphomas. Blood. 2009;113(18):4352–4361. 10.1182/blood-2008-09-179143 . 19147785

[16] Kabrun N, Bühring HJ, Choi K, Ullrich A, Risau W, Keller G. Flk-1 expression defines a population of early embryonic hematopoietic precursors. Development. 1997;124(10):2039–2048. . 9169850 10.1242/dev.124.10.2039

[17] Ishida A, Murray J, Saito Y, Kanthou C, Benzakour O, Shibuya M, et al. Expression of vascular endothelial growth factor receptors in smooth muscle cells. J Cell Physiol. 2001;188(3):359–368. 10.1002/jcp.1121 . 11473363

[18] Witmer AN, Dai J, Weich HA, Vrensen GF, Schlingemann RO. Expression of vascular endothelial growth factor receptors 1, 2, and 3 in quiescent endothelia. J Histochem Cytochem. 2002;50(6):767–777. 10.1177/002215540205000603 . 12019293

[19] Fedorova A, Zobel K, Gill HS, Ogasawara A, Flores JE, Tinianow JN, et al. The development of peptide-based tools for the analysis of angiogenesis. Chem Biol. 2011;18(7):839–845. 10.1016/j.chembiol.2011.05.011 . 21802005

[20] Fujii I, Takaoka Y, Suzuki K, Tanaka T. A conformationally purified α-helical peptide library. Thetrahedron Lett. 2001;42(19):3323–3325

[21] Barbas CF 3rd, Kang AS, Lerner RA, Benkovic SJ. Assembly of combinatorial antibody libraries on phage surfaces: the gene III site. Proc Natl Acad Sci U S A. 1991;88(18):7978–7982. 10.1073/pnas.88.18.7978 . 1896445 PMC52428

[22] Takahashi N, Kakinuma H, Liu L, Nishi Y, Fujii I. In vitro abzyme evolution to optimize antibody recognition for catalysis. Nat Biotechnol. 2001;19(6):563–567. 10.1038/89320 . 11385462

[23] Fujii I, Fukuyama S, Iwabuchi Y, Tanimura R. Evolving catalytic antibodies in a phage-displayed combinatorial library. Nat Biotechnol. 1998;16(5):463–467. 10.1038/nbt0598-463 . 9592396

[24] Goshima N, Kawamura Y, Fukumoto A, Miura A, Honma R, Satoh R, et al. Human protein factory for converting the transcriptome into an in vitro-expressed proteome, Nat Methods. 2008;5(12):1011–1017. 10.1038/nmeth.1273 . 19054851

[25] Hoshino A, Kanegane H, Nishi M, Tsuge I, Tokuda K, Kobayashi I, et al. Identification of autoantibodies using human proteome microarrays in patients with IPEX syndrome. Clin Immunol. 2019;203:9–13. 10.1016/j.clim.2019.03.011 . 30951839

[26] Hojo H, Onuma Y, Akimono Y, Nakahara Y, Nakahara Y. N-Alkyl cysteine-assisted thioesterification of peptides. Tetrahedron Lett. 2007;48(1):25–28

[27] Johnson EC, Kent SB. Insights into the mechanism and catalysis of the native chemical ligation reaction. J Am Chem Soc. 2006;128(20):6640–6646. 10.1021/ja058344i . 16704265

[28] Wan Q, Danishefsky SJ. Free-radical-based, specific desulfurization of cysteine: a powerful advance in the synthesis of polypeptides and glycopolypeptides. Angew Chem Int Ed Engl. 2007;46(48):9248–9252. 10.1002/anie.200704195 . 18046687

[29] Doronina SO, Toki BE, Torgov MY, Mendelsohn BA, Cerveny CG, Chace DF, et al. Development of potent monoclonal antibody auristatin conjugates for cancer therapy. Nat Biotechnol. 2003;21(7):778–784. 10.1038/nbt832 . 12778055

[30] Hurwitz H, Fehrenbacher L, Novotny W, Cartwright T, Hainsworth J, Heim W, et al. Bevacizumab plus irinotecan, fluorouracil, and leucovorin for metastatic colorectal cancer. N Engl J Med. 2004;350(23):2335–2342. 10.1056/NEJMoa032691 . 15175435

[31] Wilke H, Muro K, Van Cutsem E, Oh SC, Bodoky G, Shimada Y, et al. Ramucirumab plus paclitaxel versus placebo plus paclitaxel in patients with previously treated advanced gastric or gastro-oesophageal junction adenocarcinoma (RAINBOW): a double-blind, randomised phase 3 trial. Lancet Oncol. 2014;15(11):1224–1235. 10.1016/S1470-2045(14)70420-6 . 25240821

[32] Holash J, Davis S, Papadopoulos N, Croll SD, Ho L, Russell M, et al. VEGF-Trap: a VEGF blocker with potent antitumor effects. Proc Natl Acad Sci U S A. 2002;99(17):11393–11398. 10.1073/pnas.172398299 . 12177445 PMC123267

[33] Mendel DB, Laird AD, Xin X, Louie SG, Christensen JG, Li G, et al. In vivo antitumor activity of SU11248, a novel tyrosine kinase inhibitor targeting vascular endothelial growth factor and platelet-derived growth factor receptors: determination of a pharmacokinetic/pharmacodynamic relationship. Clin Cancer Res. 2003;9(1):327–337. . 12538485

[34] Podar K, Tonon G, Sattler M, Tai YT, Legouill S, Yasui H, et al. The small-molecule VEGF receptor inhibitor pazopanib (GW786034B) targets both tumor and endothelial cells in multiple myeloma. Proc Natl Acad Sci U S A. 2006;103(51):19478–19483. 10.1073/pnas.0609329103 . 17164332 PMC1748251

[35] Beck B, Driessens G, Goossens S, Youssef KK, Kuchnio A, Caauwe A, et al. A vascular niche and a VEGF-Nrp1 loop regulate the initiation and stemness of skin tumours. Nature. 2011;478(7369):399–403. 10.1038/nature10525 . 22012397

[36] Zhao D, Pan C, Sun J, Gilbert C, Drews-Elger K, Azzam DJ, et al. VEGF drives cancer-initiating stem cells through VEGFR-2/Stat3 signaling to upregulate Myc and Sox2. Oncogene. 2015;34(24):3107–3119. 10.1038/onc.2014.257 . 25151964

[37] Bhattacharya R, Ye XC, Wang R, Ling X, McManus M, Fan F, et al. Intracrine VEGF Signaling Mediates the Activity of Prosurvival Pathways in Human Colorectal Cancer Cells. Cancer Res. 2016;76(10):3014–3024. 10.1158/0008-5472.CAN-15-1605 . 26988990 PMC4873444

[38] Zhang L, Wang H, Li C, Zhao Y, Wu L, Du X, et al. VEGF-A/Neuropilin 1 Pathway Confers Cancer Stemness via Activating Wnt/β-Catenin Axis in Breast Cancer Cells. Cell Physiol Biochem. 2017;44(3):1251–1262. 10.1159/000485455 . 29179185

