# Supplementary figures and images for: A “ligand-targeting” peptide-drug conjugate: Targeted intracellular drug delivery by VEGF-binding helix-loop-helix peptides via receptor-mediated endocytosis
Source: PLoS One. 2021 Feb 25;16(2):e0247045. doi: 10.1371/journal.pone.0247045 (PMC7906330; doi:10.1371/journal.pone.0247045)

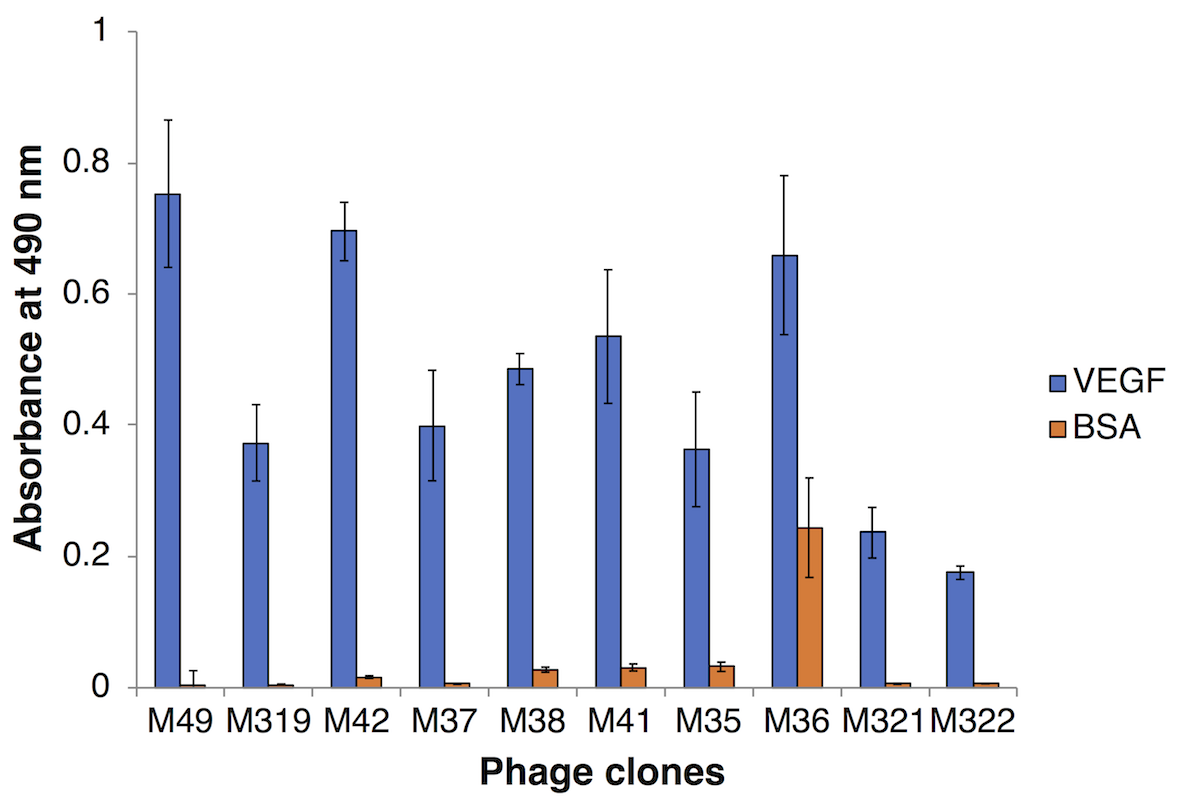

Supplement: S1 Fig — The all clones specifically bound to VEGF-A, and M49 phage clone presented the strongest binding signal to VEGF-A. The data represent the mean ± standard deviation (n = 3). (TIF) [file pone.0247045.s002.tif]

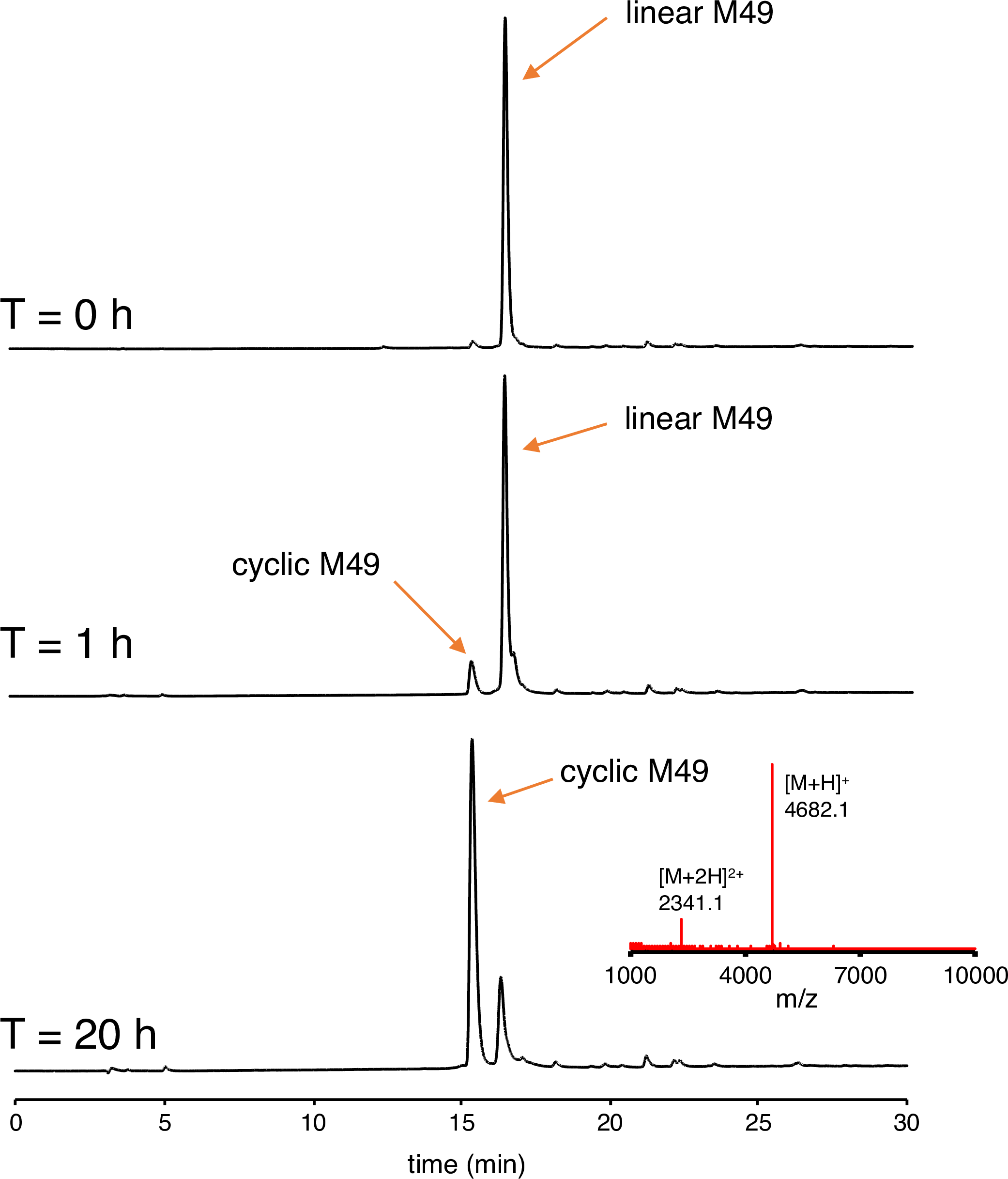

Supplement: S2 Fig — The reaction was monitored by RP-HPLC. The UV profile at 280 nm is shown. The separations were performed using a linear gradient (10–90%) of eluent B in eluent A over 30 min (eluent A = 0.1% TFA in water; eluent B = 0.08% TFA in acetonitrile). Linear M49 was converted to cyclic M49 (MALDI-TOF-MS: observed [M+H]+ 4682.1 (m/z), calculated [M+H]+ using monoisotopes 4682.6). (TIF) [file pone.0247045.s003.tif]

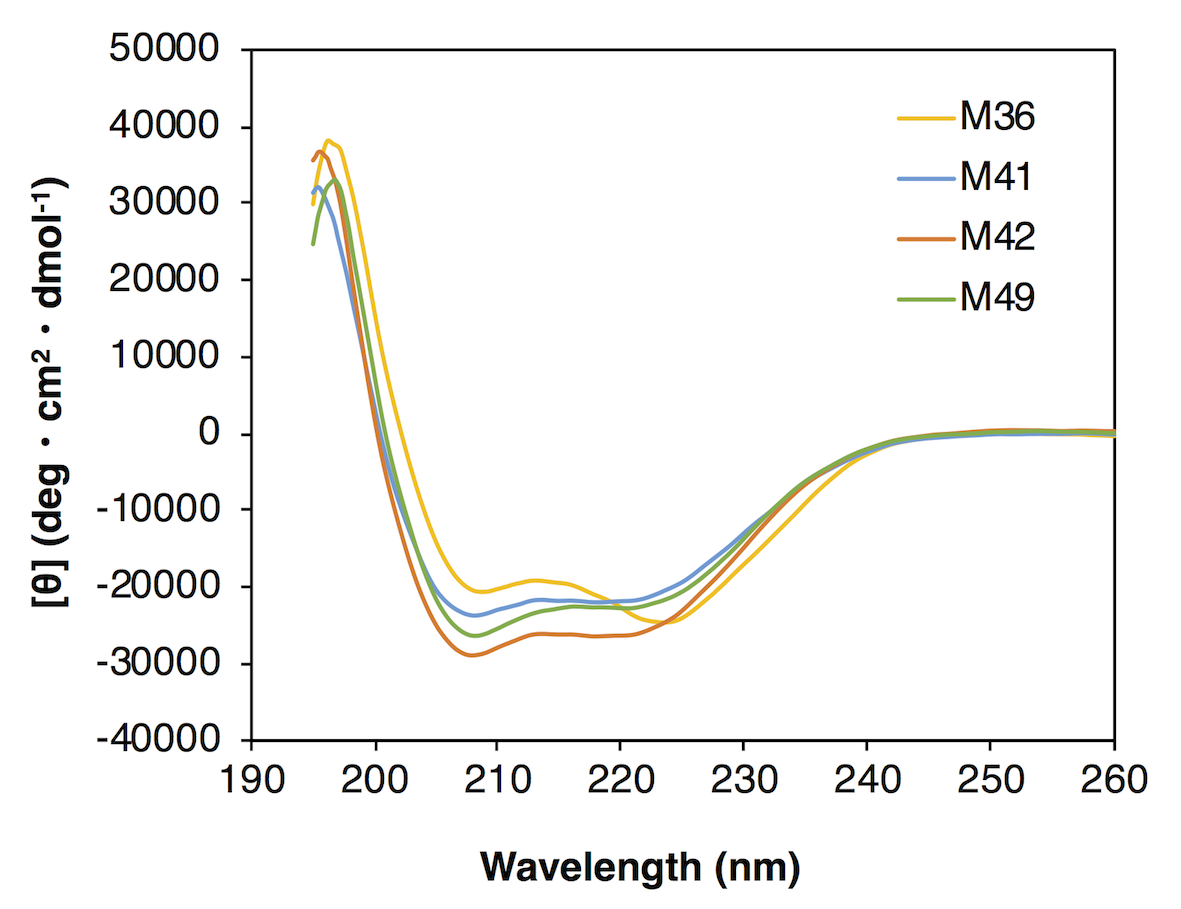

Supplement: S3 Fig — CD spectra were collected at 20°C in PBS, and the concentration of peptides was 20 μM. The synthetic peptides showed typical α-helical arrangement (negative maxima at 222 and 208 nm, positive maximum at approximately 190 nm). (TIF) [file pone.0247045.s004.tif]

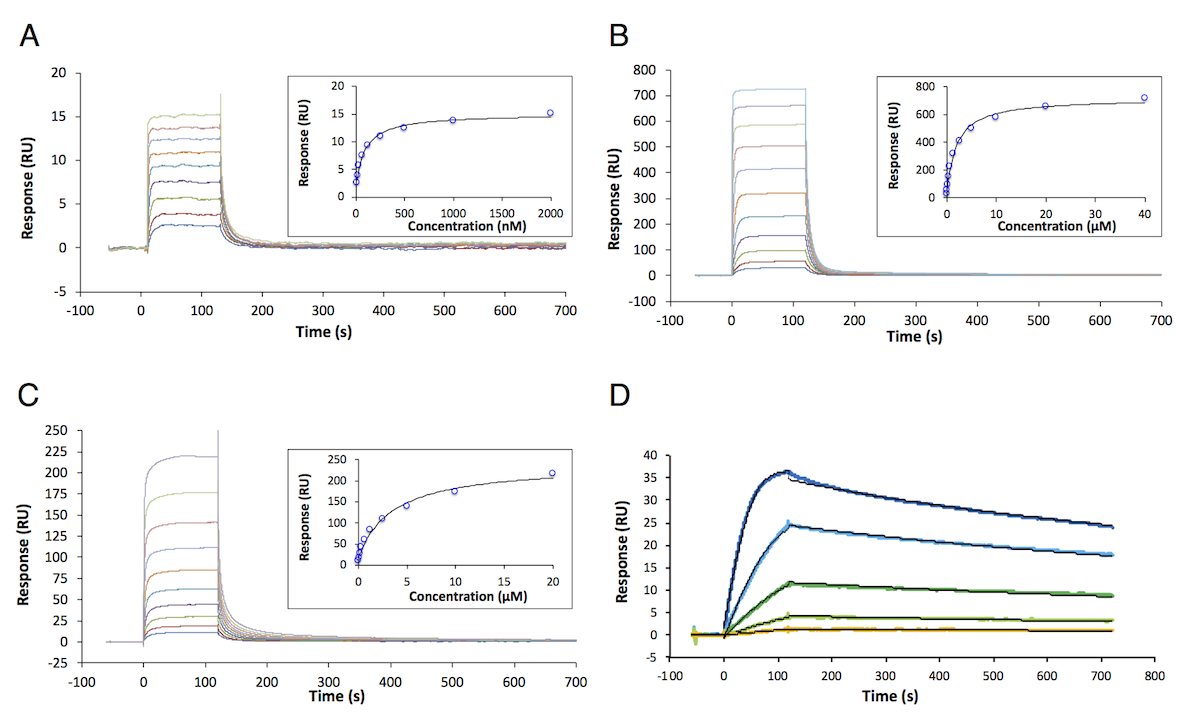

Supplement: S4 Fig — The binding affinity of (A) M36, (B) M41, (C) M42 and (D) M49 were determined with SPR. The sensorgrams and scatchard plots were presented. KD values (n = 3) were 96.9 ± 3.0 nM, 1960 ± 20 nM, 3540 ± 80 nM and 0.87 ± 0.15 nM, respectively. Sensorgrams of M49 were fitted with the 1:1 Langmuir model. ka, (9.2 ± 1.9) × 105 (1/Ms); kd, (7.6 ± 0.1) × 10−4 (1/s). (TIF) [file pone.0247045.s005.tif]

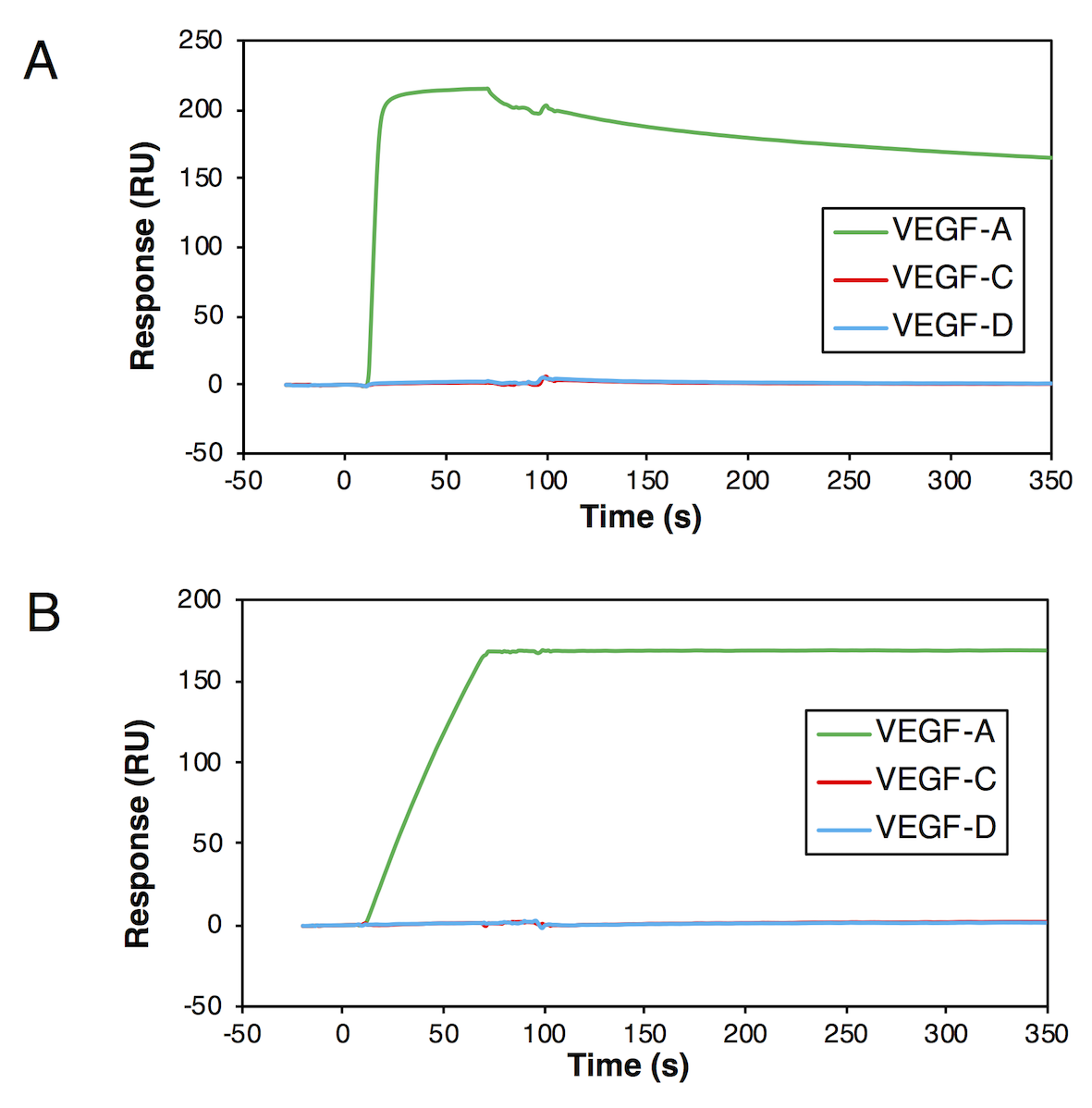

Supplement: S5 Fig — VEGF-A, -C, and -D were immobilized on CM5 sensor chip at 1000 RU by using amine coupling method. (A) The sensorgrams of peptide M49 to immobilized VEGF proteins. Peptide M49 was injected at a concentration of 1 μM, and association time was 60 seconds. (B) The sensorgrams of bevacizumab at a concentration of 100 nM. (TIF) [file pone.0247045.s006.tif]

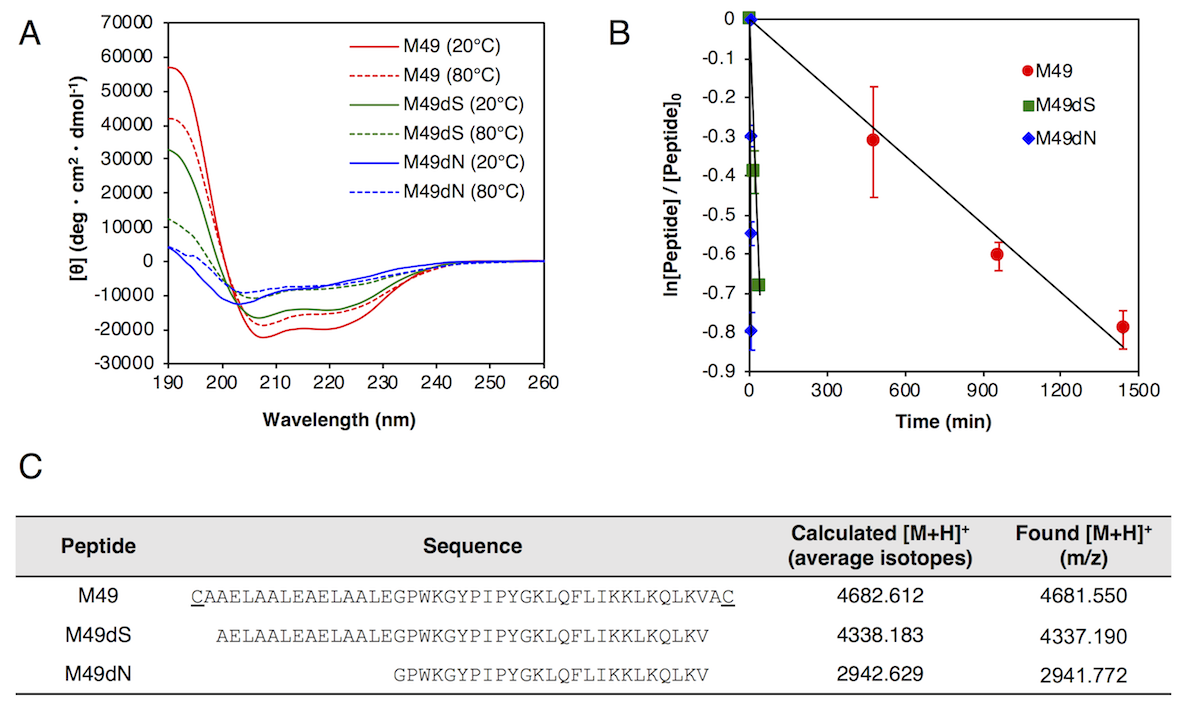

Supplement: S6 Fig — (A) CD spectra of peptide M49 and its variants at 20°C (solid line) and 80°C (dash line). (B) Tryptic stability of M49 peptide and its variants. The peptides were incubated with trypsin, and the remaining peptides were analyzed by RP-HPLC. The data represent the mean ± standard deviation (n = 3). (C) The amino acid sequence of the synthetic peptides. Underlined cysteine residues are involved in disulfide bond formation. These peptides were synthesized using Fmoc-SPPS and molecular mass was confirmed by using MALDI-TOF-MS. (TIF) [file pone.0247045.s007.tif]

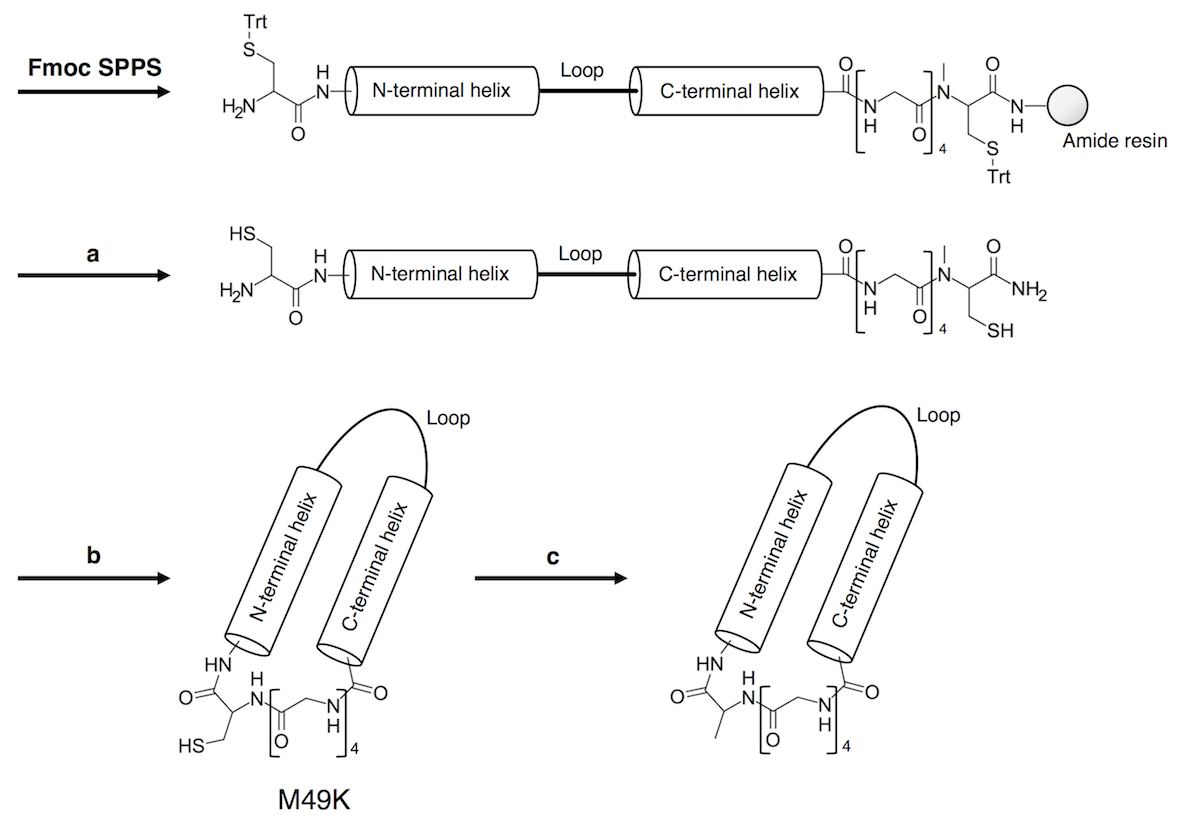

Supplement: S7 Fig — (a) 94:2.5:2.5:1 TFA/EDT/H2O/TIS, 3h, 15% yield; (b) 2 mM MPAA, 20 mM TCEP, 200 mM Na2HPO4 pH7, 12h, RT, 40% yield; (c) 200 mM VA-044, 250 mM TCEP, 40 mM Glutathione, 12h, RT, 65% yield. (TIF) [file pone.0247045.s008.tif]

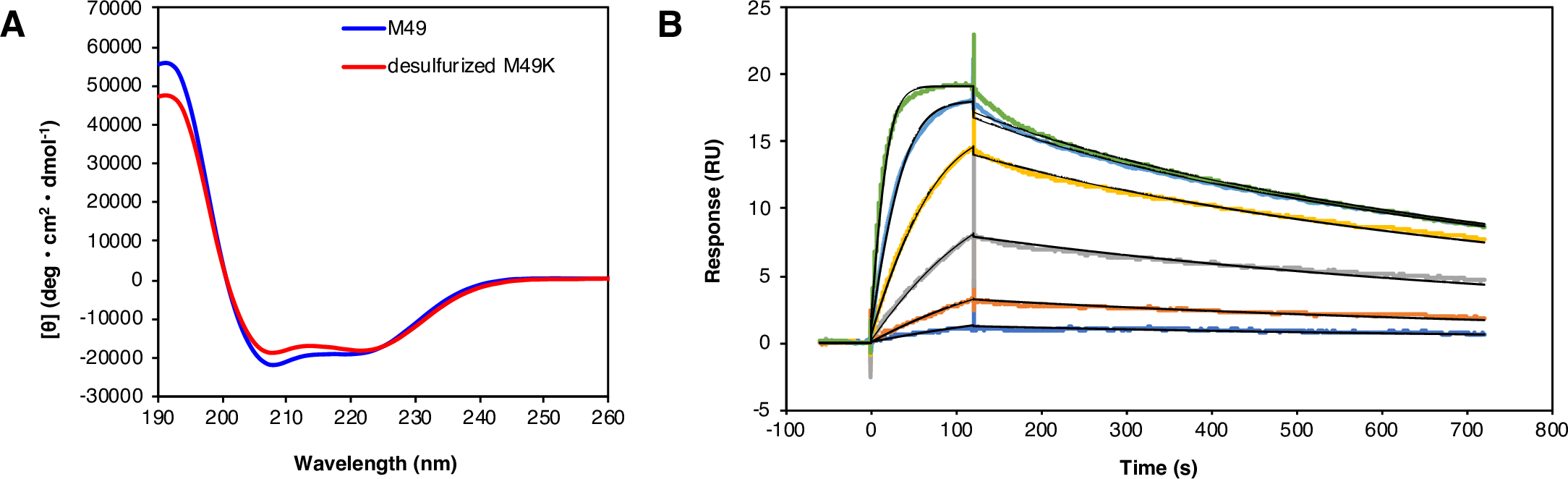

Supplement: S8 Fig — (A) CD spectra of M49 and desulfurized M49K at 20°C in 20 mM phosphate buffer (pH 7.0). (B) Sensorgrams of desulfurized M49K binding to recombinant human VEGF-A165. M49K (200–6 nM) were injected over the VEGF-A immobilized flow cell as an analyte. Flow rate, temperature, and running buffer were 3.0 μL/min, 25°C, and HBS-EP+, respectively. The binding parameters were calculated by BiacoreT200 evaluation software with the 1:1 Langmuir model. The fitting curves were indicated as black lines. KD, 4.45 ± 1.30 (nM); ka, (4.46 ± 0.71) × 105 (1/Ms); kd, (2.02 ± 0.75) × 10−3 (1/s). The data represent the mean ± standard deviation (n = 3). (TIF) [file pone.0247045.s009.tif]

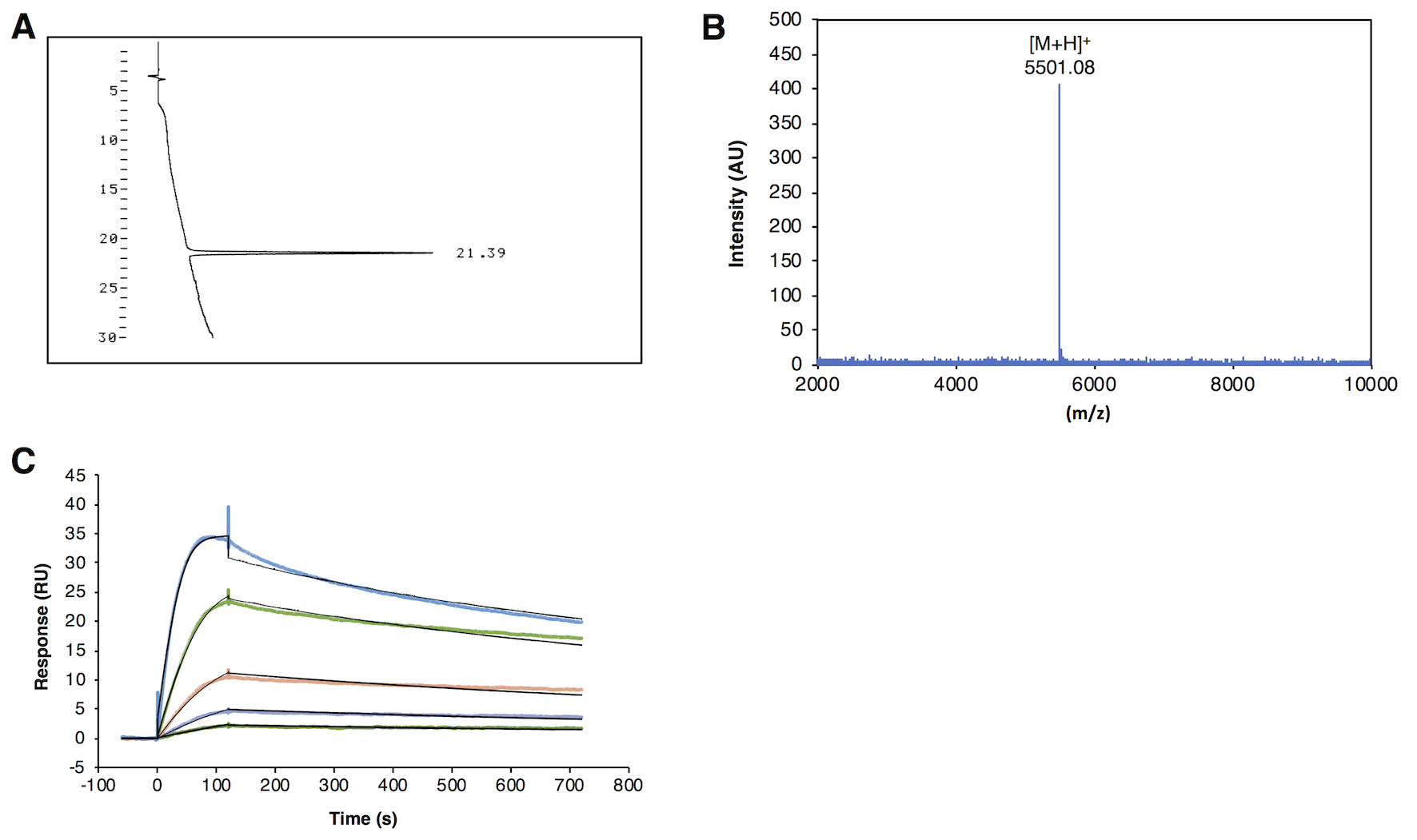

Supplement: S9 Fig — (A) Analytical HPLC profile of M49K-Cy5. Analytical HPLC was performed linear gradient (10–90%) of eluent B in eluent A over 30 min. (eluent A = 0.1% TFA in water; eluent B = 0.08% TFA in acetonitrile) on a C-18 column (250 × 4.6 mm, YMC-Pack). (B) MALDI-TOF-MS spectra of M49K-Cy5. Calculated for C261H400N58O66S3 [M+H]+: 5501.51 (average isotopes), Found [M+H]+ m/z = 5501.08 (C) Sensorgram of the M49K-Cy5 binding to VEGF-A165. M49K-Cy5 was injected over the VEGF immobilized flow cell as an analyte (400–25 nM) at 25°C. The binding parameters were calculated by BiacoreT200 evaluation software with the 1:1 Langmuir model. The fitting curves were indicated as black lines and KD value was 5.5 nM. (TIF) [file pone.0247045.s010.tif]

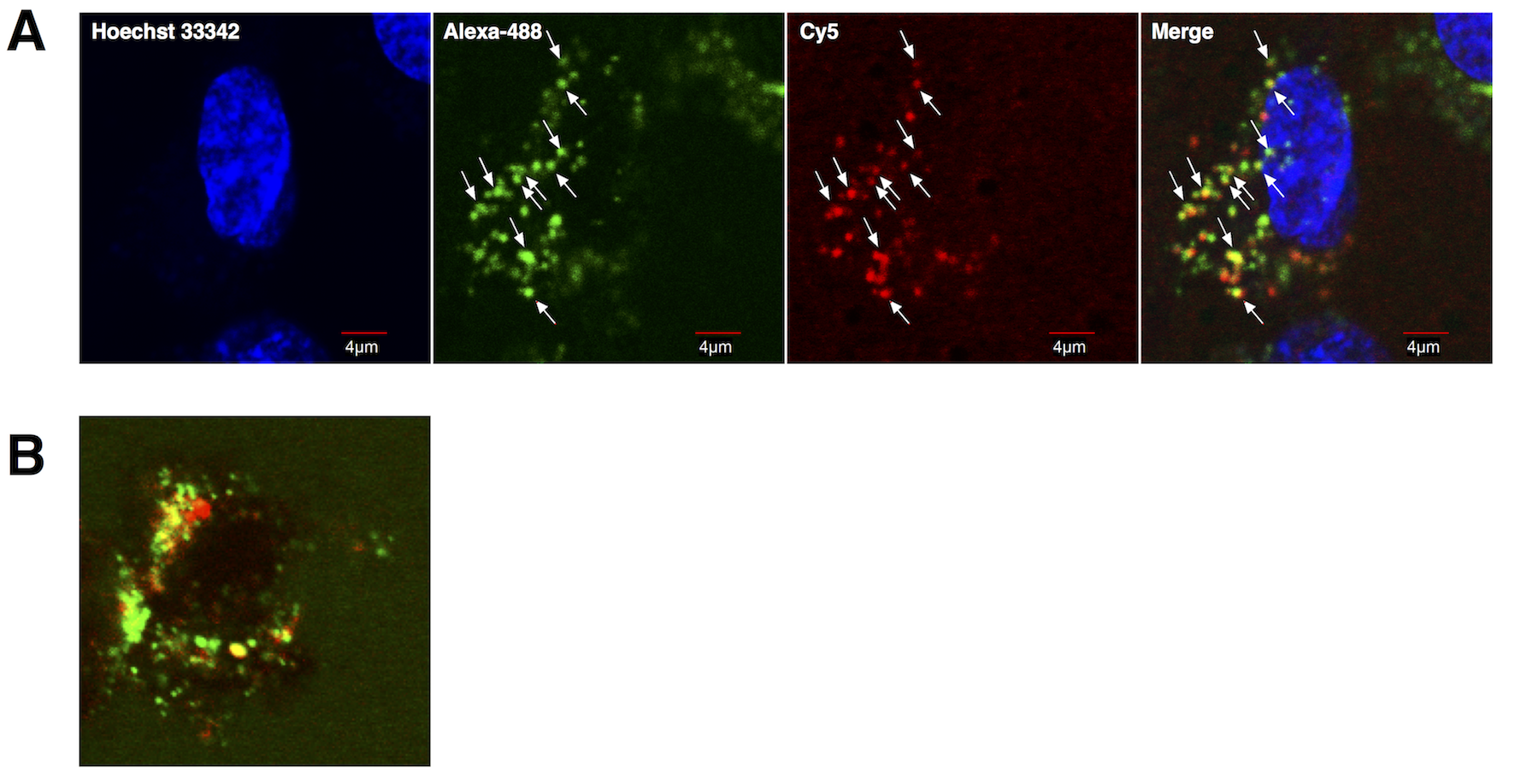

Supplement: S10 Fig — (A) HUVECs were treated with M49K-Cy5 (500 nM) and VEGF-Alexa488 (100 nM) for 6 hours at 37°C. Cell nuclei were stained by Hoechst 33342 before imaging. White arrows indicate co-localized vesicles. (B) An enlarged image of Fig 3c in the main text. (TIF) [file pone.0247045.s011.tif]

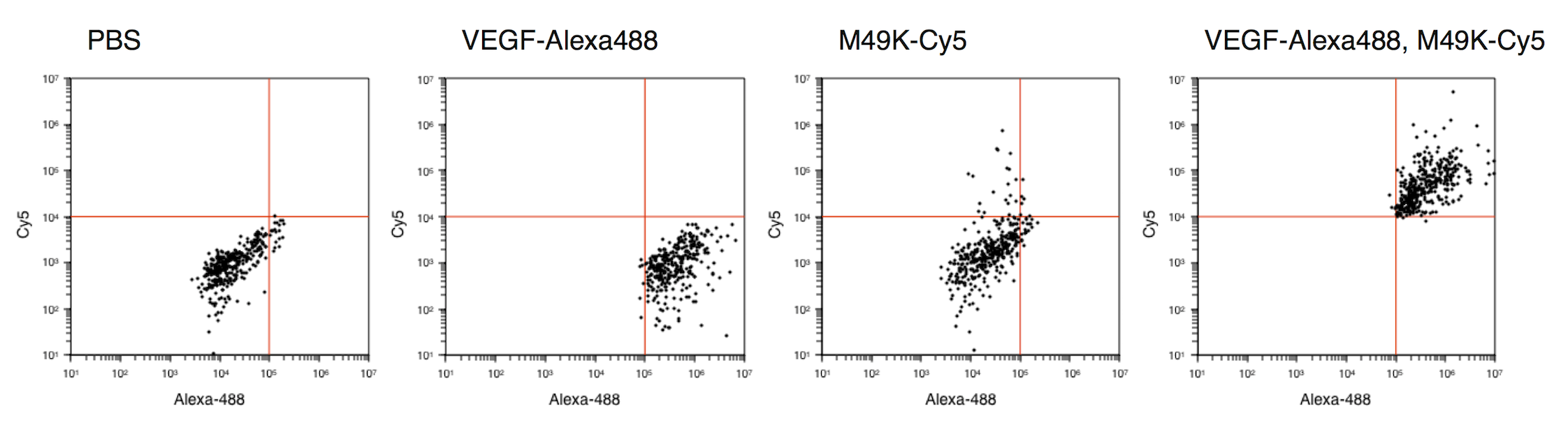

Supplement: S11 Fig — HUVECs were incubated for 6 hours with different solutions: VEGF-Alexa488 (100 nM), M49K-Cy5 (500 nM), and mixture of VEGF-Alexa488 (100 nM) and M49K-Cy5 (500 nM). PBS treated cells served as negative control. (TIF) [file pone.0247045.s012.tif]

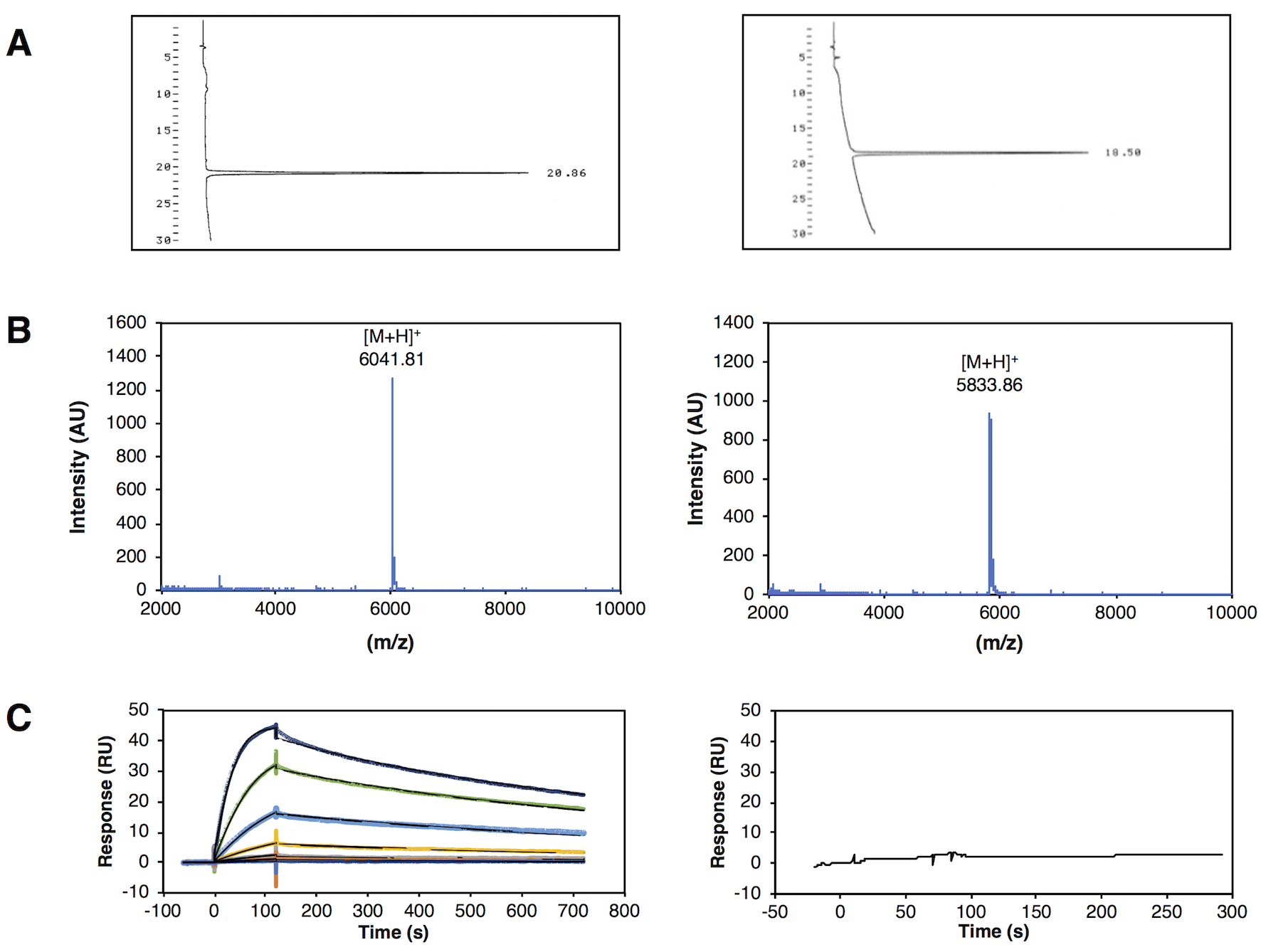

Supplement: S12 Fig — (A) Analytical HPLC profile of purified PDCs. Analytical HPLC was performed linear gradient (10–90%) of eluent B in eluent A over 30 min. (eluent A = 0.1% TFA in water; eluent B = 0.08% TFA in acetonitrile) on a C-18 column (250 × 4.6 mm, YMC-Pack). (B) MALDI-TOF-MS spectra of PDCs. M49K-vcMMAE: Calculated for C290H461N65O72S [M+H]+: 6042.22 (average isotopes), Found [M+H]+ m/z = 6041.81, M49Kmut-vcMMAE: Calculated for C276H452N64O71S [M+H]+: 5834.99 (average isotopes), Found [M+H]+ m/z = 5833.86 (C) Sensorgrams of the PDCs binding to VEGF-A165. M49K-vcMMAE was injected over the VEGF immobilized flow cell as an analyte (1000–16 nM). The binding parameters were calculated by BiacoreT200 evaluation software with the 1:1 Langmuir model. The fitting curves were indicated as black lines. KD value was 30 nM. M49Kmut-vcMMAE was injected at a concentration of 1000 nM. (TIF) [file pone.0247045.s013.tif]

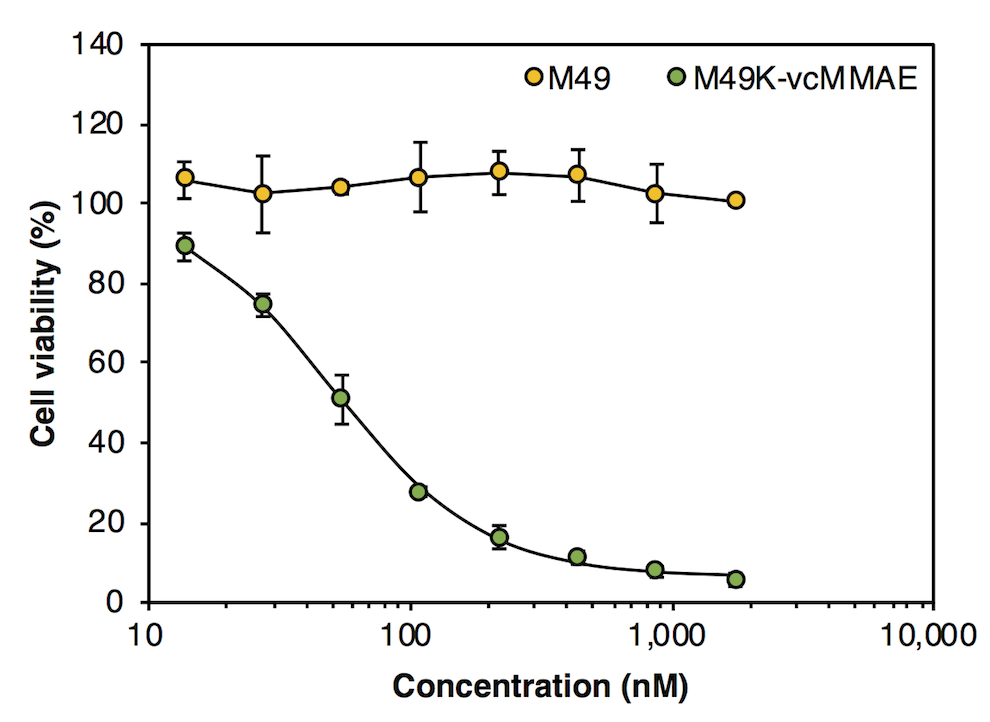

Supplement: S13 Fig — The IC50 value of M49K-vcMMAE is 50 nM. The data represent the mean ± standard deviation (n = 3). (TIF) [file pone.0247045.s014.tif]

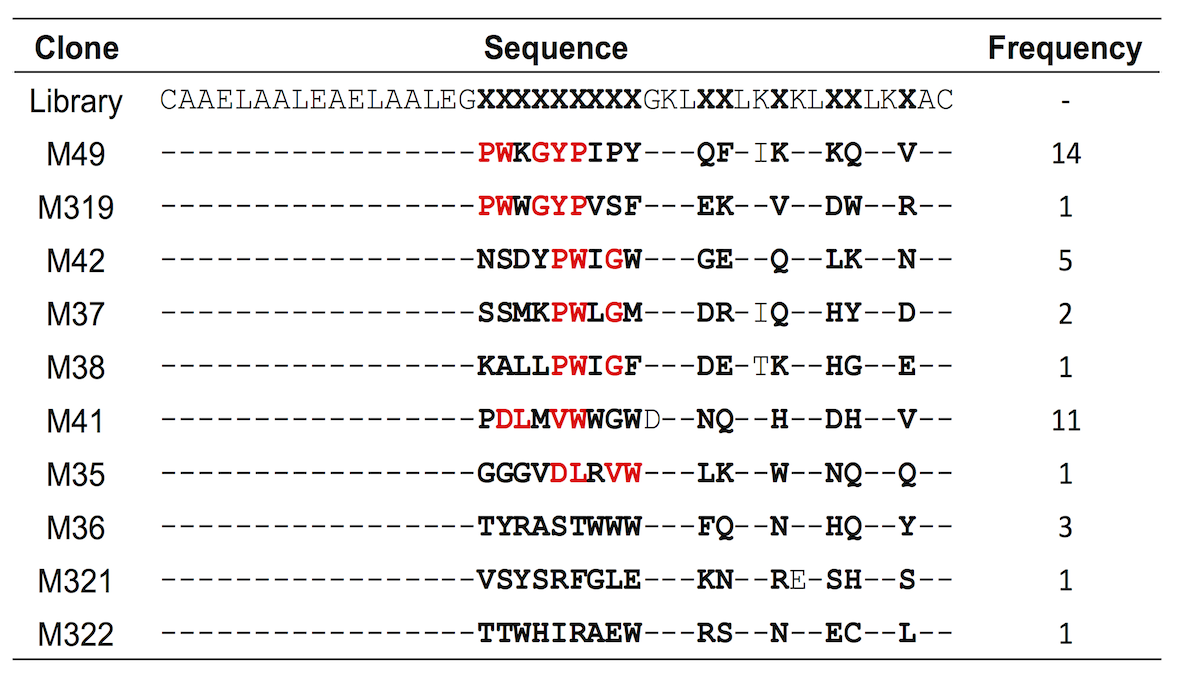

Supplement: S1 Table — After 4 rounds of the screening, we obtained 10 HLH peptide clones. The consensus sequences were displayed in red, and the numbers of identified clones were showed as frequency. (TIF) [file pone.0247045.s015.tif]

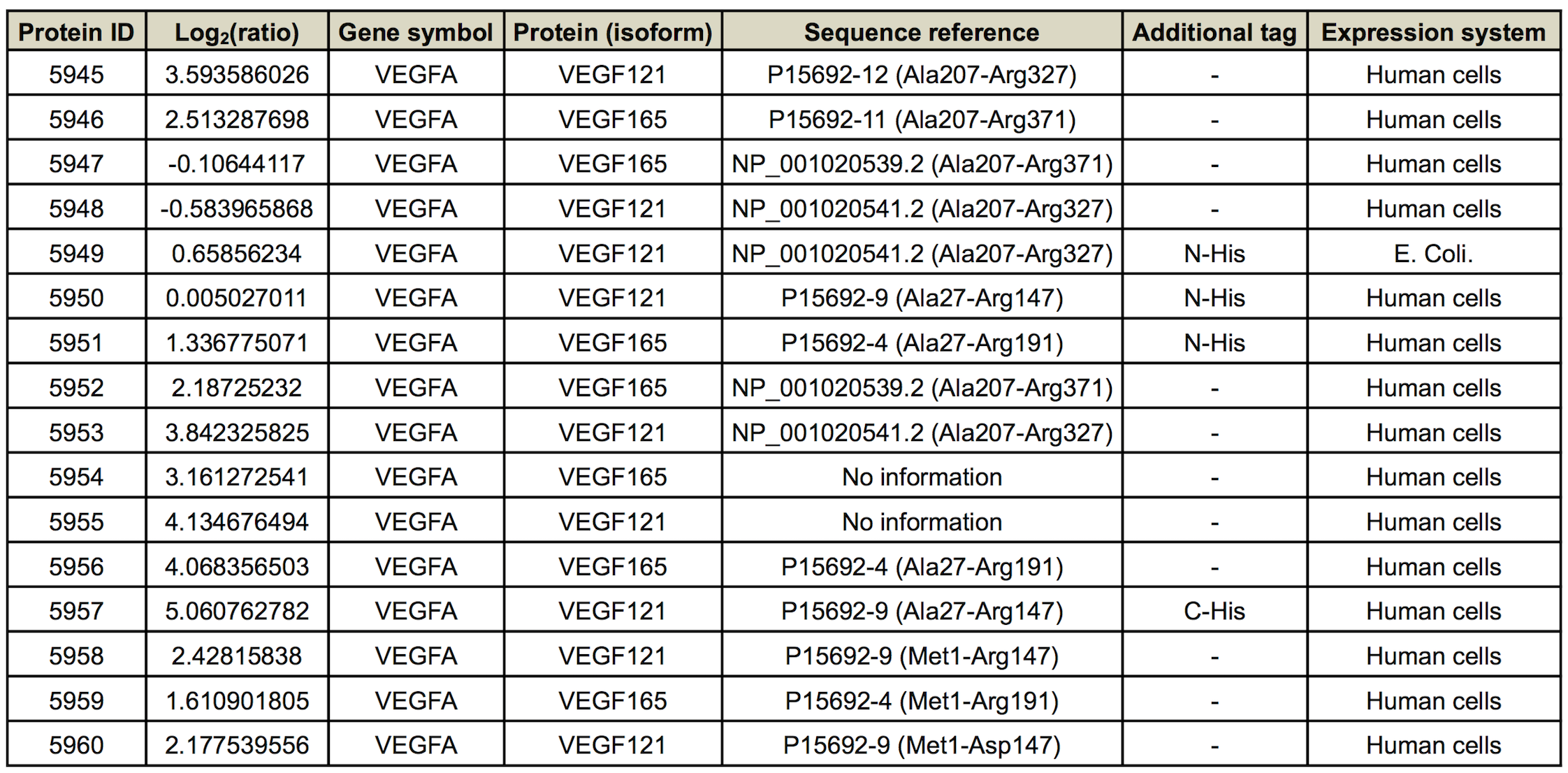

Supplement: S2 Table — (TIFF) [file pone.0247045.s016.tiff]
